# Transcriptomic analysis after SARS-CoV-2 mRNA vaccination reveals a specific gene signature in low-responder hemodialysis patients

**DOI:** 10.3389/fimmu.2025.1508659

**Published:** 2025-04-30

**Authors:** Simone Lucchesi, Giorgio Montesi, Jacopo Polvere, Fabio Fiorino, Gabiria Pastore, Margherita Sambo, Marialetizia Lusini, Francesca Montagnani, Annalisa Ciabattini, Francesco Santoro, Guido Garosi, Donata Medaglini

**Affiliations:** ^1^ Laboratory of Molecular Microbiology and Biotechnology, Department of Medical Biotechnologies, University of Siena, Siena, Italy; ^2^ Department of Medicine and Surgery, LUM University “Giuseppe Degennaro”, Bari, Italy; ^3^ Department of Medical Biotechnologies, University of Siena, Siena, Italy; ^4^ Infectious and Tropical Diseases Unit, University Hospital of Siena, Siena, Italy; ^5^ Nephrology, Dialysis, and Transplantation Unit, University Hospital of Siena, Siena, Italy

**Keywords:** biomarkers, transcriptomics, hemodialysis, SARS-CoV2 vaccine, EPO (Erythropoietin)

## Abstract

**Introduction:**

Individuals with comorbidities, such as chronic kidney disease and hemodialysis patients (HDP), are particularly susceptible to severe COVID-19 and to its complications. Furthermore, their immune response to vaccines is impaired, requiring tailored vaccination strategies. In this study, we investigated through transcriptomic profiling the immune response heterogeneity of HDP vaccinated with two doses of mRNA BNT162b2 vaccine.

**Methods:**

Transcriptomic analyses were conducted in peripheral blood mononuclear cells (PBMC) collected from HDP and healthy controls (HC) before and 7 days after each dose. The HDP were stratified into high- and low-responders based on their humoral response after the second dose.

**Results:**

Significant differences in gene expression related to B cell abundance and regulation, CD4 T cell proliferation, and inflammation pathways were observed at baseline and day 7 between HDP-low responders and HC, while the HDP high-responders displayed an intermediate expression profile for these genes.

**Discussion:**

Results were consistent with the known immunologic alterations occurring in HDP cohorts related to lymphopenia, chronic inflammation, and dysregulated proliferation of CD4+. Our analyses identified an early transcriptional signature correlated with a diminished immune response in HDP low-responders, highlighting the importance of conducting a characterization of immunocompromised cohorts.

## Introduction

The SARS-CoV-2 pandemic has had a profound global impact since its outbreak. Advanced age, male gender, and underlying comorbidities such as hypertension, diabetes, immunodeficiency, and kidney diseases are primary risk factors for severe outcomes (1). Individuals with chronic kidney disease (CKD) and those requiring dialysis are particularly susceptible to SARS-CoV-2 infection, often experiencing a severe course of COVID-19 and higher mortality (2).

CKD is commonly associated with immunodeficiency, leading to reduced vaccine efficacy against various pathogens, including HBV and *Streptococcus pneumoniae* (3–5). Immunological alterations in CKD patients include reduced B cell counts and altered T cell phenotypes (6, 7). CDK patients and in particular hemodialysis patients (HDP) exhibit a diminished immune response to vaccines, necessitating modifications in vaccination strategies such as adjusting the dosage or dosing interval to enhance immunogenicity (8).

As observed for other immunocompromising pathologies (9–11), the humoral immune response following SARS-CoV-2 vaccination in HDP is heterogeneous and delayed compared to healthy subjects (12), with lower antibody levels and faster antibody decline (13). Notably, the humoral response in HDP after two vaccine doses is lower compared to other fragile CKD cohorts, such as patients undergoing peritoneal dialysis (14) or CKD patients not undergoing hemodialysis (15) and they require three or four doses to reach antibody titers comparable to the general population (16).

Moreover, cell-mediated immunity, including T cell-mediated cytokine induction (17) and antigen-specific CD4+ and CD8+ T cell responses (18), is also diminished in HDP compared to healthy controls (HC) and other vulnerable cohorts (19).

Systems vaccinology and transcriptomics have emerged as powerful techniques for identifying gene signatures associated with immune activation or suppression, providing insights into the biological pathways involved in vaccine immunogenicity (20–22).

In this study, we aimed to identify early differences in gene expression and pathways predictive of vaccine immune responsiveness, and characterize at transcriptional level the immune response in HDP who received two doses of the BNT162b2 mRNA vaccine. Immunological data, including anti-Spike IgG titers and ACE2/RBD binding inhibition, were used to stratify HDP into high- and low-responders.

## Methods

### Study cohort

Blood samples were obtained from adult HDP and from adult HC, who received two doses of mRNA vaccine Comirnaty BNT162b2 (Pfizer-BioNTech), 3 weeks apart, according to national schedules. A total of 20 HDP were enrolled in the context of the PatoVac_COV study, while 9 HCs were enrolled in the IMMUNO_COV study. All participants were recruited at the Infectious and Tropical Diseases Unit and at Nephrology, Dialysis, and Transplantation Unit, Azienda Ospedaliera Universitaria Senese (Siena, Italy), where they provided written informed consent before joining the study. Inclusion criteria were age ≥ 18 years and adherence to the COVID-19 vaccination campaign (for both studies), hemodialysis treatment (only for PatoVac_COV study). Exclusion criteria were pregnancy, withdrawal of consent or refusal to participate (for both studies), clinical problems for collecting additional blood samples beyond the amount required for routine care and participation to other clinical studies (for PatoVac_COV study), being affected by any immunocompromising condition (congenital, acquired, or drug-related; for IMMUNO_COV study).

### Study approval

The studies were performed in compliance with all relevant ethical regulations and the protocol was approved by local Ethical Committee for Clinical experimentation of Regione Toscana Area Vasta Sud Est (CEAVSE; protocol code 19479 PatoVac_COV v1.0 of 03 Mar 2021, approved on 15 Mar 2021 for HDP and protocol code 18869 IMMUNO_COV v1.0 of 18 Nov 2020, approved on 21 Dec 2020 for HC).

### Sample and data collection

Venous blood samples were collected in heparin-coated blood tubes (BD Vacutainer) at the baseline (day 0), at days 7, 21 after the first vaccine dose, 7 days after the second dose (day 28). PBMCs were isolated by density-gradient sedimentation, using Ficoll-Paque (Lymphoprep, Stemcell technologies, USA). Approximately 2-3x10^6^ PBMCs were mixed in 1 ml of QIAzol Lysis Reagent (Qiagen, Netherlands), incubated for 1 h at room temperature (RT) and then stored at -80°C for RNA extraction. Plasma samples were stored at -80°C. Clinical parameters, including age, gender, comorbidities, albumin levels, and mobility, were collected to calculate a health state score using the Couchoud method (23). Although Couchoud method is validated for patients older than 75 years, clinicians routinely use it in real life for prognostic purposes and we therefore assume it as a suitable score for the statistical analyses.

Weekly erythropoietin (EPO) dosage was also collected, both before the first vaccine dose and as an average over the six months preceding the initial dose, for subsequent integrative analyses.

Clinical data collection and management were carried out using the software REDCap (Research Electronic Data Capture, Vanderbilt University, TN, USA).

### Enzyme-linked immunosorbent assay

Anti-Spike IgG production after SARS-CoV-2 mRNA vaccination was tested by ELISA, as previously described (24). Maxisorp microtiter plates (Nunc, Denmark) were coated overnight at 4°C with wild type SARS-CoV-2 full spike protein (S1+S2 ECD; Sino Biological), at a concentration of 1 μg/ml in PBS (Sigma-Aldrich). Plates were blocked at room temperature (1h, RT) with blocking solution (5% skimmed milk powder, 0.05% Tween 20, 1 × PBS). Plasma samples were titrated in two-fold dilutions in diluent buffer (3% skimmed milk powder, 0.05% Tween 20, 1 × PBS) and added in duplicate. After 1 h at RT, anti-human IgG horseradish peroxidase (HRP)-conjugated antibody (diluted 1:6,000; Southern-Biotech, Birmingham, AL, USA) was added for 1 h at RT. Plates were developed with 3,3’,5,5’-Tetramethylbenzidine (TMB; Thermo Fisher Scientific) for 10 min at RT, followed by the addition of 1 M stop solution (Thermo Fisher Scientific). Absorbance at 450 nm was immediately measured on a Multiskan FC Microplate Photometer (Thermo Fisher Scientific). A WHO international positive control (plasma from a vaccinated donor; NIBSC) and negative control (plasma from an unvaccinated donor; NIBSC) were added in duplicate to each plate as internal control for reproducibility. Antibody endpoint titers were calculated as the reciprocal of the last sample dilution that doubled the OD450 value compared to the background.

### Surrogate virus neutralization test

ACE2/RBD binding inhibition was tested with a SARS-CoV-2 surrogate virus neutralization test (sVN) kit (cPass™ SARS-CoV-2 Neutralization Antibody Detection Kit; Genscript, Piscataway, NJ, USA) according to the manufacturer protocol, as previously described (25). Briefly, plasma samples, positive and negative controls were diluted 1:10, mixed with HRP-RBD (wild type variant RBD) and incubated for 30 min at 37°C. Mixtures were added to an ACE2-coated 96-well plate and incubated for 15 min at 37°C. After washing, TMB solution was added and the plate was developed for 15 min at RT. The reaction was quenched by adding 1M stop solution, and the OD450 was read with Multiskan FC Microplate Photometer (Thermo Fisher Scientific). Results were expressed as follows: percentage inhibition = (1 − sample OD value/negative control OD value) × 100. Inhibition values ≥30% were considered as positive results, and values <30% as negative results, as established by Tan et al. (26) and indicated by the manufacturer.

### RNA extraction, library preparation and sequencing

PBMCs frozen in QIAzol were thawed at RT and RNA was extracted with a phase separation using 200 µl chloroform (15s vortex, then incubated 3 minutes at RT, then centrifuged 15’ 12,000 × *g* at 4°C). Aqueous phase was transferred in 500 µl isopropanol, vortexed, incubated at RT for 10’ and centrifuged for 15 minutes at 12,000 × *g* at 4°C. The supernatant was discarded and the pellet washed with 1 ml of 75% ethanol. The pellet was suspended in 350 µl RLT solution (Qiagen) and total RNA was extracted using the RNeasy mini kit on a QIAcube instrument (Qiagen). RNA quantification was performed using Qubit 4 Fluorometer (Thermofisher) with Qubit RNA High Sensitivity assay kit (Invitrogen). RNA integrity was assessed using the 2100 Bioanalyzer instrument (Agilent) with the Agilent RNA 6000 Nano Kit (Agilent).

50 ng of total RNA were used as a template for the preparation of sequencing libraries using the Illumina Stranded Total RNA Prep Ligation with Ribo-Zero Plus (Illumina) following manufacturer’s instructions. Libraries were quantified with NEBNext^®^ Library Quant Kit for Illumina^®^ (New England Biolabs) and with the Qubit DNA High Sensitivity kit (Invitrogen). Average library size was measured using Agilent DNA 1000 Kit (Agilent) on the 2100 Bioanalyzer instrument (Agilent). A paired-end sequencing was performed using the NovaSeq 6000 System (Illumina) on a S4 flowcell (200 cycles).

### Pre-processing and data analysis

Pre-processing steps were performed in a Linux environment using FASTQC v0.11.9 and MultiQC v1.13 for the quality control. Trimming and removal of low-quality reads was performed with Trimmomatic v0.39 using the following parameters: SLIDINGWINDOW 4:5, MINLEN:36. STAR v2.7.3 was used to align reads to reference Grch38 human genome while htseq v.2.0.2 to count the reads of annotated genes.

Data analysis was conducted on R v.4.2.2, an open-source coding platform. For all analyses, the whole dataset was split into four smaller datasets each containing only samples from one time point. Gene counts were filtered to exclude low or not expressed genes applying a filter of at least 1 count per million (CPM) in at least 10 libraries, then DESeq2 package v1.36.0 (27) was used to normalize gene expression and the vst function (variance stabilizing transformation) was used to transform and export normalized counts (28).

Non-linear dimensionality reduction techniques were applied to each normalized and z-score transformed dataset using Uniform Manifold Approximation and Projection (29) with UMAP R package v0.2.10, in a umap-learn configuration, to better visualize differences between the cohorts. Quantitative separation was assessed using the unsupervised K-means clustering algorithm with the kmeans function (setting centers = 3) on the dimensionality-reduced space and was compared with experimental group labels in terms of accuracy (number of correctly classified as a fraction of total samples).

Differential gene expression (DGE) analysis was performed following DESeq2 function pipeline (27), p-values were adjusted for multiple testing employing Benjamini-Hochberg method, and significant genes were selected as p.adjust<0.05. Enrichment analysis was conducted with tmod v0.50.11 library (30) using CERNO test (31) to assess the enrichment in Blood Transcription Modules (BTMs) (32).

Modular gene co-expression Network analysis was carried out with CEMiTool v1.20.0 (33), able to perform such analysis in an unsupervised manner by automatically detecting best *ad hoc* soft thresholding power BETA. For co-expression analysis with CEMiTool, a unique dataset including samples from all time points was used. Module enrichment analysis, relying on hypergeometric tests, was conducted to assess the enrichment of each identified module in BTMs. A score for each module was also calculated as the mean z-score of genes within each module and statistical differences among mean z-scores of various groups were computed using non-parametric Mann-Whitney test.

Feature selection was performed with DaMirSeq v2.12.0 (34), by applying a backward variable elimination in Partial Least Squares method (bve-PLS) and a multivariate filter technique. Portions of features used for the analysis were selected at the Elbow Point of the graph Variance explained. Only genes selected by both CEMiTool and DamirSeq feature selections were kept in the final output. Further enrichment analyses were performed using gene ontology (35) and Web-based Cell-type-Specific Enrichment Analysis of Genes (WebCSEA) (36) databases.

### Statistics

The statistical significance of ELISA titers, ACE2/RBD inhibition, and clinical parameters were assessed with Mann-Whitney U test. Fisher exact test was used for binary variables. DGE analysis was performed following DESeq2 function pipeline (27), p-values were adjusted for multiple testing employing Benjamini-Hochberg method, and significant genes were selected as p.adjust<0.05. The statistical significance of CEMiTool scores were assessed with Mann-Whitney U test. All tests were two-tailed.

Analyses were performed using GraphPad Prism v9 (GraphPad Software, CA, USA) and in R programming environment.

## Results

In this study, we conducted a transcriptomic profiling of PBMCs from 20 HDP and 9 HC, who received the BNT162b2 SARS-CoV-2 mRNA vaccine. Gene expression analysis was performed on total PBMCs at baseline (day 0), days 7 and 21 after the first vaccine dose, and 7 days after the second dose (day 28) and transcriptomic data were integrated with immunological parameters. This comprehensive approach aimed to provide a robust comparison of the immune responsiveness observed in HDP versus HC. Furthermore, the spike-specific IgG response, including antibody titers and ACE2/RBD binding inhibition, was analyzed at day 28 and utilized to stratify the HDP cohort in the transcriptomic analysis. The experimental design and analysis workflow are summarized in **Supplementary Figure 1**, while clinical and demographic data are reported in **Supplementary Table 1**.

### Immunological profile of HDP and vaccine antigen-specific antibody response

**Figure 1:**
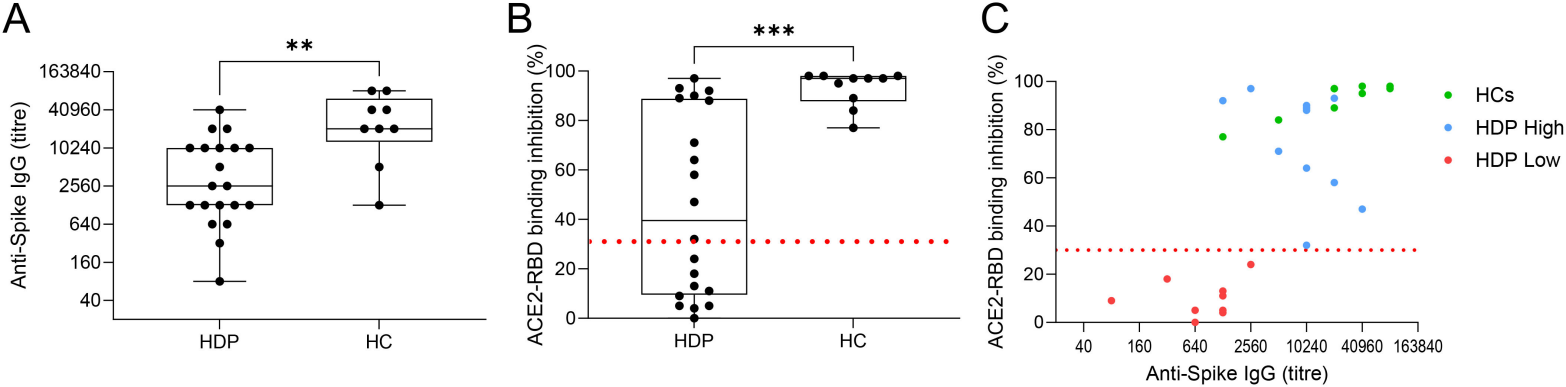
HDP cohort stratification. **(A)** Spike-specific IgG analyzed by ELISA in plasma collected at day 28 after the first dose of the BNT162b2 mRNA vaccine in hemodialysis patients (HDP) and healthy controls (HC). Antibody titers are expressed as the reciprocal of the dilution of sample reporting an OD value double respect to the background. Data are shown as box and whisker plot where box represents the interquartile range (IQR), horizontal line represents the median value and whiskers represent higher and lower values. Individual values are reported as dots. **(B)** Surrogate virus neutralization assay (sVN) performed on plasma collected at day 28. Data are reported as ACE2/RBD binding inhibition percentage with box and whisker plots. A threshold (dotted red line) was placed at 30% inhibition percentage to discriminate between positive and negative samples in HDP and HC. **(C)** Bivariate plot showing Anti-Spike IgG titers (X axis) and ACE2/RBD binding inhibition percentage (Y axis) in HC (in green) and HDP-high and -low (blue and red, respectively). The non-parametric Mann-Whitney Test was used to assess significant differences (*p-value<0.05, **p-value<0.01, ***p-value<0.001).

Analysis of the spike-specific antibody response, measured seven days after the second vaccine dose, revealed a significantly lower response in HDP compared to HC (**Figure 1A**). To assess the functionality of spike-specific IgG antibodies in inhibiting ACE2/RBD binding, we employed a surrogate virus neutralization test (sVN). The results were reported as percentages of ACE2/RBD inhibition, with values ≥30% considered positive (**Figure 1B**). Nine out of 20 HDP patients (45%) had a neutralizing activity lower than 30%, while all HC were above this threshold (**Figure 1B**). By plotting the antibody titers against the inhibition activity, the HDP cohort was stratified into two groups: (i) HDP who tested positive for the sVN test and showed spike-specific IgG levels comparable to those of HC (HDP-high group), and (ii) HDP negative for the sVN test and with lower IgG levels compared to the HCs (HDP-low group) (**Figure 1C**).

### Transcriptomic profiles of hemodialysis patients in response to mRNA vaccination

**Figure 2:**
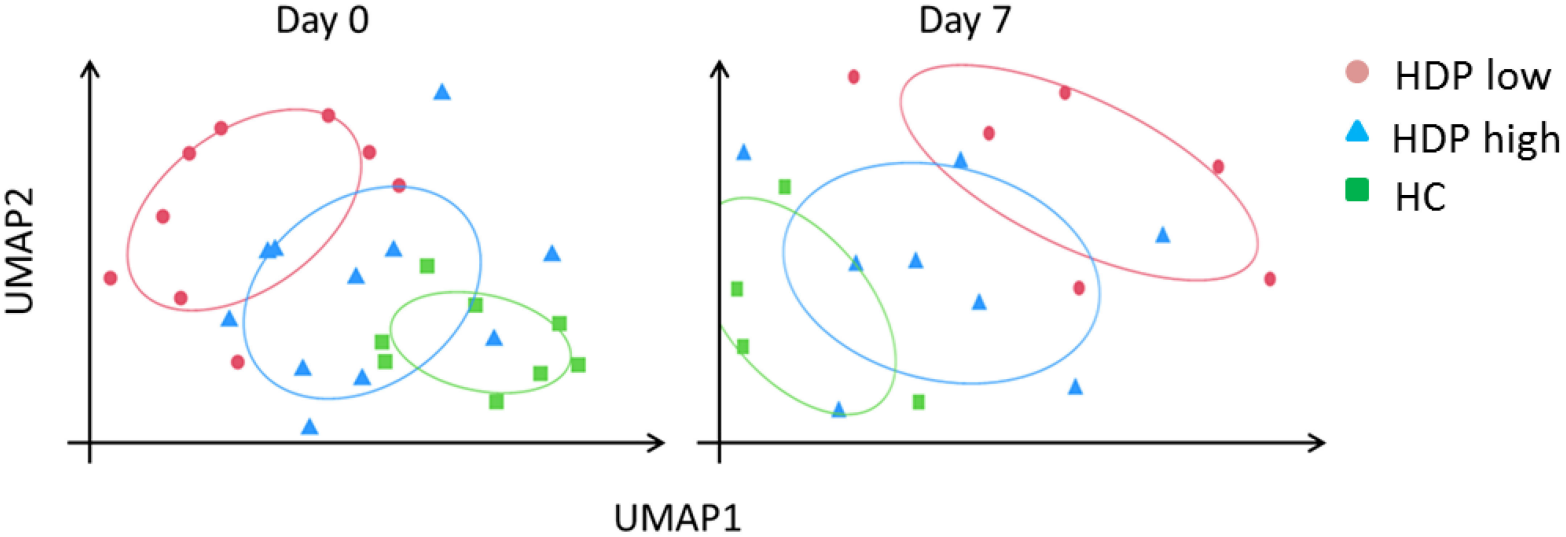
UMAP dimensionality reduction at day 0 and day 7. Normalized and z-score transformed gene counts were dimensionally reduced using the UMAP (Uniform Manifold Approximation and Projection) algorithm. Each dot represents a sample in the two new surrogate dimensions (UMAP1 and UMAP2). Each color represents an experimental group. Colored ellipses represented 75% confidence intervals.

Transcriptome sequencing was performed to characterize differences at the baseline level and in response to mRNA vaccination in the HDP-low and HDP-high groups, compared to HC. Unsupervised dimensionality reduction analysis was performed, at each time point, with the Uniform Manifold Approximation and Projection (UMAP) algorithm. At day 0 and 7 days after the first vaccine dose, UMAP revealed a distinct separation between HDP-high and HDP-low samples, as well as between the hemodialysis cohort and HC (**Figure 2**). However, when the analysis was conducted at day 21 (pre-second dose) and day 28 (7 days after the second dose), the main separation was between HDP and HC, with lower or no separation between the HDP-high and HDP-low samples (**Supplementary Figure 2**). k-Means unsupervised clustering was used to confirm the better separation in the UMAP dimensionality-reduced space among the three cohorts at days 0 and 7 compared with the days 21 and 28 (**Supplementary Table 2**).

To examine gene expression differences among the three cohorts at each time point, a differential gene expression (DGE) analysis was performed using DESeq2 and results are summarized in **Table 1** and **Supplementary Table 3**. A high number of statistically significant DEGs was observed at each time point when comparing HCs with HDP-low, while a lower number of DEGs was detected between HDP-high responders and HC, particularly at day 0 and day 7, confirming UMAP results where HDP-high were clustered near HC (**Figure 2**).

**Table 1:** Differential gene expression analysis.

Day	Contrast	Up-regulated genes	Down-regulated genes	Unaffected genes
Day 0	HDP-low vs HDP-high	21	9	19564
	HDP-high vs HC	82	10	18743
	HDP-low vs HC	1524	1813	16257
Day 7	HDP-low vs HDP-high	64	51	16440
	HDP-high vs HC	9	1	16166
	HDP-low vs HC	632	546	18416
Day 21	HDP-low vs HDP-high	283	57	19254
	HDP-high vs HC	893	184	18517
	HDP-low vs HC	1403	626	17565
Day 28	HDP-low vs HDP-high	300	98	17677
	HDP-high vs HC	408	551	18635
	HDP-low vs HC	1541	1120	16933

**Figure 3:**
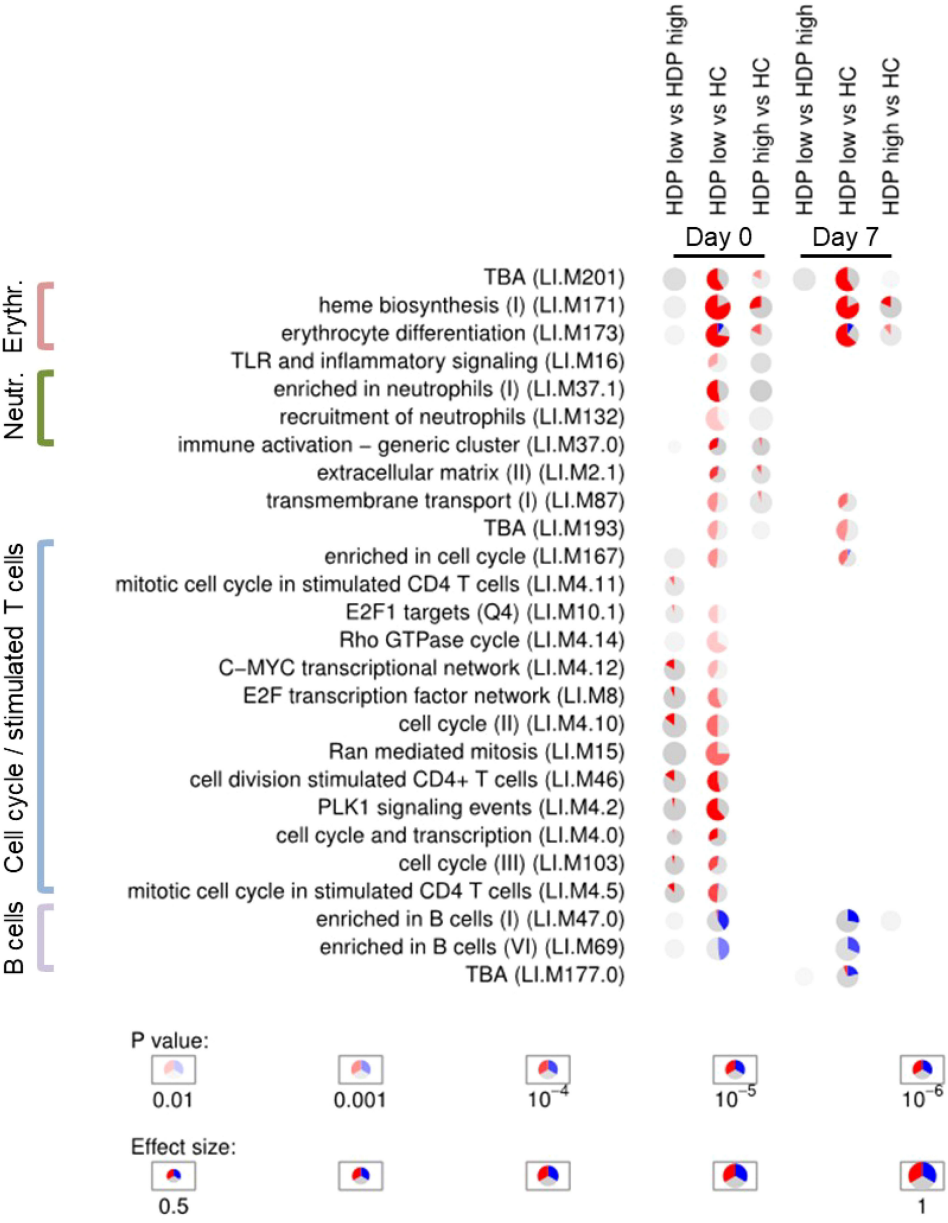
Enrichment analysis. Activation of blood transcription modules by BNT162b2 vaccination in healthy controls (HC) and hemodialysis patients stratified in high- and low-responders groups (HDP-high and HDP-low, respectively). Each column represents a comparison. Only comparisons from day 0 and day 7 are shown. Activation of modules was tested using tmod CERNO test on the false discovery rate (FDR)-ranked lists of genes generated by DESeq2. Rows indicate different blood transcription modules, which were significantly (FDR < 0.01) activated in at least one comparison. Each module is represented as a pie plot in which the proportion of significantly upregulated and downregulated genes is shown in red and blue, respectively. The grey portion of the pie represents genes that are not significantly differentially regulated according DGE analysis. The significance of module activation is proportional to the color intensity of the pie, while the effect size (Area Under the Curve) is proportional to its size.

Enrichment analyses were conducted to assess the enrichment of blood transcription modules (BTMs) (32) for each DGE analysis in order to obtain an overview of the functional roles of the genes identified through DGE analysis. A selection of statistically significant enriched BTM at days 0 and 7 is displayed in **Figure 3**, full results of enrichment analysis are reported in **Supplementary Figure 3**. At all the time points, pie plots clearly show the upregulation of modules involved in erythrocyte differentiation, cell cycle, and heme biosynthesis when comparing both HDP-low and HDP-high with HC. Additionally, modules associated with T cells, cell cycle, and proliferation were significantly upregulated in HDP, especially in HDP-low, at day 0 and day 21. Conversely, B cell modules were downregulated in HDP-low compared to HC at each time point. The comparison between HDP-low and HDP-high at day 7 resulted in the significant enrichment of two unannotated modules (LI.M177.0 and LI.M201); of those, LI.M177.0 included 4 genes belonging to *GIMAP* family, which is involved in B and T cells survival (37, 38).

### Co-expression analysis reveals differences in transcriptional response following BNT162b2 mRNA vaccination in HDP-low

To further investigate the transcriptional perturbation induced by BNT162b2 vaccination, a modular gene co-expression network analysis was conducted using the unsupervised algorithm CEMiTool. In our dataset, 1438 genes were selected as relevant by unsupervised gene filtering and assigned to 11 modules (named M1 to M11) of co-expressed genes (**Table 2**). The full gene list is reported in **Supplementary Table 4**, while **Table 2** reports the number of genes within each CEMiTool module, the top 5 co-expressed/representative hub genes, and the top statistically significant enriched BTMs.

**Table 2:** CEMiTool modules. For each module, the number of genes, the hub genes and the enrichment analysis are reported.

Module	Number of genes	Hub genes	Enriched BTMs
M1	417	RNF10, RIOK3, MKRN1, GSPT1, ASCC2	heme biosynthesis (I) (M171) [9], erythrocyte differentiation (M173) [7], transcription regulation in cell development (M49) [11], heme biosynthesis (II) (M222) [6], enriched in cell cycle (M167) [6], enriched in membrane proteins (M124) [6], enriched in activated dendritic cells (II) (M165) [7], antiviral IFN signature (M75) [5]
M2	188	C19orf38, BST1, LRRC25, GLT1D1, SIGLEC9	enriched in monocytes (II) (M11.0) [59], cell cycle and transcription (M4.0) [48], Monocyte surface signature (S4) [23], TLR and inflammatory signaling (M16) [12], myeloid cell enriched receptors and transporters (M4.3) [10], immune activation - generic cluster (M37.0) [32], formyl peptide receptor mediated neutrophil response (M11.2) [6)
M3	178	TUBB1, CTTN, SH3BGRL2, PDE5A, ARHGAP6	platelet activation - actin binding (M196) [11], platelet activation & blood coagulation (M199) [9], cell adhesion (M51) [12], enriched in myeloid cells and monocytes (M81) [11], cell movement, Adhesion & Platelet activation (M30) [8], G protein mediated calcium signaling (M159) [3]
M4	150	NAMPT, TRIB1, B3GNT5, CXCL8, LUCAT1	putative targets of PAX3 (M89.0) [9], chemokines and inflammatory molecules in myeloid cells (M86.0) [9], enriched for TF motif TTCNRGNNNNTTC (M172) [5], putative targets of PAX3 (M89.1) [5], cell cycle and growth arrest (M31) [5], growth factor induced, enriched in nuclear receptor subfamily 4 (M94) [5], AP-1 transcription factor network (M20) [5], myeloid, dendritic cell activation via NFkB (I) (M43.0) [5], enriched in activated dendritic cells/monocytes (M64) [5], CCR1, 7 and cell signaling (M59) [4]
M5	136	CD22, FCRL1, LINC00926, FCRL2, NIBAN3	enriched in B cells (I) (M47.0) [32], enriched in B cells (II) (M47.1) [22], enriched in B cells (VI) (M69) [15], B cell surface signature (S2) [21], plasma cells & B cells, immunoglobulins (M156.0) [12], enriched in B cells (III) (M47.2) [8]
M6	79	KCNJ15, LIMK2, TNFRSF10C, LRRC4, DGAT2	enriched in neutrophils (I) (M37.1) [23], immune activation - generic cluster (M37.0) [19], TLR and inflammatory signaling (M16) [8],recruitment of neutrophils (M132) [4], Monocyte surface signature (S4) [5]
M7	67	IGKV4-1, IGKV3-20, IGKC, MZB1, IGKV1-5	plasma cells, immunoglobulins (M156.1) [10], plasma cells & B cells, immunoglobulins (M156.0) [7], enriched in B cells (III) (M47.2) [2], enriched in B cells (IV) (M47.3) [2]
M8	64	CEACAM6, CEACAM8, CAMP, ABCA13, BPI	immune activation - generic cluster (M37.0) [31], extracellular matrix (II) (M2.1) [9], extracellular matrix, complement (M140) [4], enriched in membrane proteins (M124) [4], cell division (stimulated CD4+ T cells) (M46) [3]
M9	40	ENSG00000283994, POU5F2, ENSG00000271204, LINC01619, ENSG00000286786	none
M10	37	NR4A2, NR4A3, TNFAIP3, CREM, ZNF331	putative targets of PAX3 (M89.0) [4], putative targets of PAX3 (M89.1) [3], growth factor induced, enriched in nuclear receptor subfamily 4 (M94) [3], chemokines and inflammatory molecules in myeloid cells (M86.0) [2]
M11	36	UTY, USP9Y, KDM5D, DDX3Y, TXLNGY	chromosome Y linked (M240) [5], NK cell surface signature (S1) [5], enriched in NK cells (KIR cluster) (M61.1) [3], enriched in NK cells (receptor activation) (M61.2) [3], enriched in NK cells (II) (M61.0) [3]

The number of genes within each BTM is reported in square brackets.

**Figure 4:**
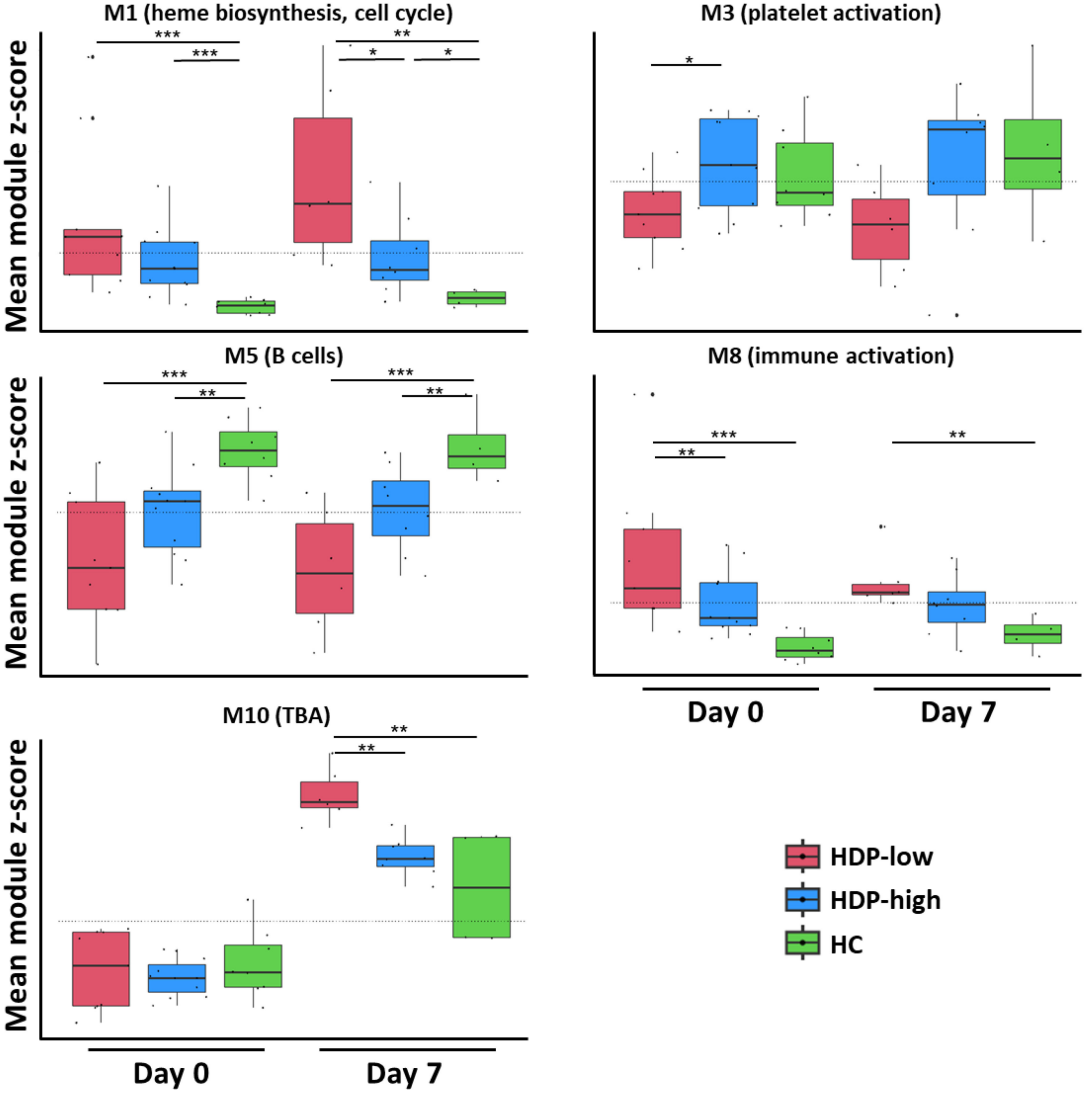
CEMiTool analysis. A CEMiTool analysis was performed on the normalized gene counts to group genes with similar expression and characterize different expression patterns. Eleven co-expressed modules were identified; here, 5 modules were selected for their significant differential abundance at early time points (day 0 and day 7). Each module was represented as a mean z-score of the genes included in the module. The module score in different groups at different time points was represented as a box and whiskers plot where the box represents the IQR, the horizontal line represents the median value, and whiskers represent higher and lower values. Individual values are reported as dots. Dashed lines represent the mean value for each module. The non-parametric Mann-Whitney Test was used to assess significant differences (*p-value<0.05, **p-value<0.01, ***p-value<0.001).

The expression level of the modules, calculated as the mean z-score of genes within each module, was used to assess statistically significant differences. Module M10 displayed a distinct trend in mean z-scores at day 7, capable of differentiating the response between HDP-high and HDP-low (**Figure 4**). However, it showed poor enrichment in BTMs, mainly associated with a few genes of the PAX3 transcriptional factor putative targets. Among the 37 genes of module M10 there are 4 *DUSP* family members (*DUSP2*, *DUSP4*, *DUSP5*, and *DUSP8*). Gene ontology (GO) analysis detected enrichment in B cell homeostasis (GO:0001922; *HIF1A* and *TNFAIP3*) and negative regulation of B cells (GO:0050869; *TNFAIP3*, *TNFRSF21*, and *SAMSN1*). The B cell-related enriched M5 was significantly higher in HCs compared to HDP-low at all time points (**Figure 4** and **Supplementary Figure 4**), while M7, enriched in immunoglobulins and plasma cells modules, was up-regulated in HC at day 28 (**Supplementary Figure 4**). M3, enriched in platelet-related genes, blood coagulation, and cell adhesion modules, was significantly higher at day 0 in HDP-high compared to HDP-low, while M1, a large, heterogeneous module including genes related to erythrocytes and heme biosynthesis, cell cycle, dendritic cells, and IFN signature, and M10 were significantly up-regulated in HDP-low at day 7 (**Figure 4**). M8, enriched in a generic immune activation module and extracellular matrix related genes, exhibited a similar pattern, with lower expression in HC at all time points.

### Integration with clinical data

Since we detected a baseline enrichment of red blood cells-related modules in HDP, especially in HDP-low, we investigated whether there were differences in erythropoietin (EPO) dosage between HDP-high and HDP-low groups. EPO is commonly administered to hemodialysis patients to increase hemoglobin levels, given the dialysis-induced anemia (39). While the therapeutic efficacy of EPO is typically observed over several months of treatment, changes in gene expression can occur within a few days (40). We compared the weekly EPO dosage before the first vaccine dose and the average weekly dosage in the six months before the first dose; age was also included in the analysis. The average EPO dosage was significantly higher in HDP-low compared to HDP-high (**Supplementary Figures 5A, B**), while there was no significant difference when comparing the age between the groups (**Supplementary Figure 5C**). We then speculated that the EPO dosage before vaccination could be a proxy of the overall clinical state of the HDP cohort and therefore we calculated the health state score of all the patients with the Couchoud method, which takes into account different clinical and demographic parameters, and is proportional to the three-month risk of mortality (23). Analysis of the health state score indicated that the HDP-low cohort had a significantly higher three-month mortality risk (median: 12.8%, IQR 6-14.2%) compared to the HDP-high cohort (median: 3.0%, IQR 1.9-4.2%), confirming that the HDP-low group exhibited a more unfavorable clinical profile before vaccination (**Supplementary Figure 5D**). All clinical variables analyzed are reported in **Supplementary Table 5**.

### Feature selection identifies an early gene signature of immunogenicity in HDP cohorts

**Figure 5:**
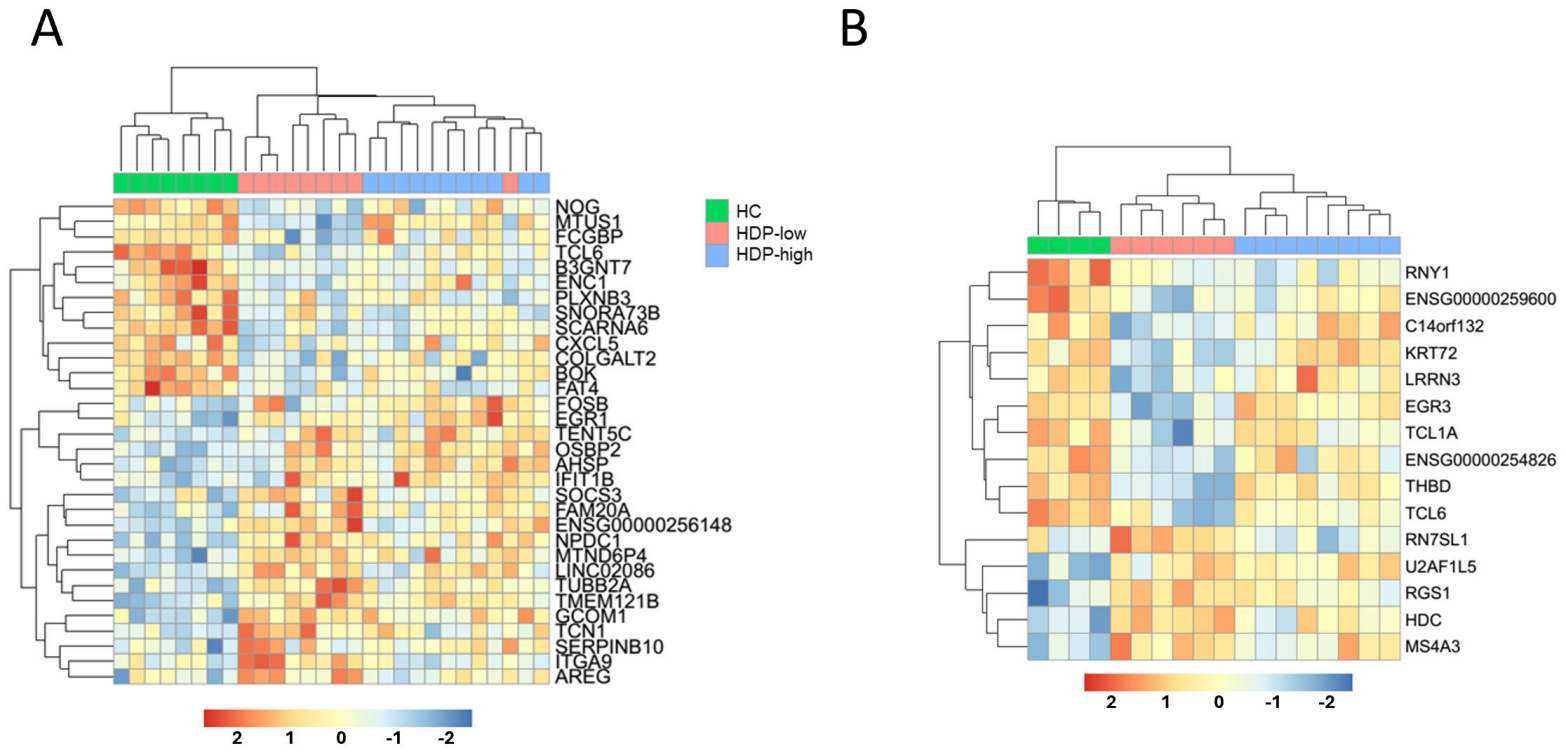
Feature selection to identify a predictive signature of immunogenicity. DaMiRseq algorithm was used to identify a gene signature able to discriminate among cohorts. The output was filtered to include only genes previously selected by the CEMiTool analysis. Normalized expression levels of **(A)** the 32 predictive genes identified for day 0, and **(B)** the 15 predictive genes for day 7, visualized as a heatmap. Samples and genes are ordered by hierarchical clustering according to gene expression levels, the upper line shows sample group.

Feature selection was conducted with the DaMirSeq package to identify an early gene signature that could distinguish the different cohorts. To refine the gene signature, we only included genes that were selected by both CEMiTool and DaMiRseq. The results of the feature selection for day 0 and day 7 are reported in **Figure 5**, revealing the identification of a gene signature (32 genes for day 0 and 15 for day 7) that exhibited discriminatory potential between HC and HDP groups, as well as between high- and low-responders within the HDP cohort. A distinct expression pattern of the selected genes between the HDP-low and HC cohorts is evident, with a subset of genes showing higher expression in the HDP-low cohort and another subset of genes exhibiting higher expression in the HC cohort while the HDP-high cohort displayed an intermediate expression level.

Some of the day 0 genes had previously been identified as differentially expressed in studies on dialysis and kidney diseases (*NOG*, *FCGBP*, *CXCL5*, *FAT4*, *SOCS3*, *ITGA9*, *AREG*, *TUBB2A*, *OSBP2*, *ENC1*) (41–49). On the other hand, among the 15-gene signature selected for day 7, only EGR3 had previously been implicated in immune functions in patients with end-stage renal disease (50). Enrichment analysis performed with WebCSEA database identified 4 of the genes upregulated in HDP-low (*U2AF1L5*, *RGS1*, *HDC*, and *MS4A3*) as expressed in mast cells.

## Discussion

This study investigated the gene expression patterns following vaccination against SARS-CoV-2 with the BNT162b2 mRNA vaccine in HDP. Although transcriptomic analyses of immune gene pathways elicited by SARS-CoV-2 vaccination in healthy subjects have been previously published (22, 51), there is currently a lack of data for HDP. A recent study by Chang et al. (52) identified a positive correlation between an upregulation in interferon-related genes 2 days after second dose and antibody titers in HDP. Similar correlations were also observed in HC vaccinated against SARS-CoV-2 and other pathogens (53, 54).

In our analysis, using a data-driven approach, we first stratified patients according to their serological response to vaccination, we then focused on assessing differences between low- and high-responders at each time point, both before and 7 days after the first and second doses. These time points allowed for the analysis of both the innate and adaptive responses, as well as baseline gene expression levels that could be predictive of a later serological response.

The main differences between HDP-high and -low responders were detected at early time points (days 0 and 7). B cell-related genes consistently exhibited lower expression levels in the dialyzed subjects. This difference was particularly pronounced when comparing HDP-low with HCs. These findings align with previous observations of B cell lymphopenia in hemodialysis patients (6) and with the correlation between low levels of B cells and increased risk of mortality from COVID-19 (55). HDP exhibited an upregulation of cell cycle-related genes in CD4+ T cells when compared with HC, particularly at day 0 and day 21. These modules also showed higher expression in the HDP-low compared with both HDP-high and HC. Previous studies reported differences in T cell frequency and phenotype between HDP and healthy cohorts (6, 56). The activation of cell cycle-related modules may be attributed to impaired proliferation of activated CD4+ T cell subsets, as observed in patients with end-stage renal failure (57). CEMiTool modules M1 and M8, enriched in cell cycle, dendritic cell signature, and IFN signature (M1) and generic immune activation and cell cycle (M8), were highly expressed especially in the HDP-low, possibly reflecting higher levels of chronic inflammation, which is commonly observed in CKD and hemodialysis (58, 59). M1 was also enriched in heme biosynthesis and erythrocyte-related genes: alterations in erythrocyte counts are well-known in HDP, and EPO treatment is commonly used to address anemia in these individuals. Interestingly, 32 genes from the M1 module were reported to be up-regulated by EPO treatment (40). We assessed the EPO dosage and found it significantly higher in HDP-low, suggesting a potential correlation with immune response.

Thrombocytopenia, a condition observed in some hemodialysis patients (60), could be reflected by a low expression at each time point in HDP-low of M3 module, enriched in platelet modules. Day 7 revealed a statistically significant modulation of the unannotated module LI.M177.0, which included a high number of *GIMAP* family genes, involved in the survival of B and T lymphocytes (37, 38). Even if not significant in DGE analysis (FDR>0.05), all these genes showed a down-modulation in HDP-low. The correlation of down-modulation of *GIMAP* family genes with the more severe lymphopenia in HDP-low cohort should be further investigated. The CEMiTool M10 module was upmodulated in HDP-low at day 7 and it is enriched in GO terms related to the negative regulation of B cells (GO:0001922 and GO:0050869), suggesting a dysregulation of these pathways in HDP-low. Notably, the *TNFRSF21* gene within M10 acts as a proapoptotic gene in lymphocytes (61). B and T cell lymphopenia and T cell activation in HDP has been associated with increased apoptosis due to uremia (6, 57), but no research to our knowledge has explored the negative regulation of B cells following vaccination in HDP. Our findings indicate a potentially dysregulated response of these pathways after the primary response in low-responder subjects. M10 also included four members of the *DUSP* family which are involved in processes ranging from inflammation to adaptive immune responses and IgE-mediated mast cell degranulation and are dysregulated in different kidney diseases (62). Interestingly, our analyses revealed a hyper activation of some members of the *DUSP* family at day 7 in the HDP-low cohort. *CXCR4*, included in M10, plays a vital role in numerous biological processes. It acts as a receptor for extracellular ubiquitin (63), which is upregulated in HDP (64). In our dataset, *UBB* (Ubiquitin B, included in M1 module) was indeed upregulated in HDP-low individuals. This gene is also upregulated upon EPO treatment (40). Limited data are available regarding the effect of EPO treatment on the immune response to vaccination, however it influences both innate and adaptive immune responses (65). Some studies suggest that EPO may enhance the humoral response in fragile subjects vaccinated with influenza or hepatitis B vaccines (66, 67). Our data contrasts with these findings. However, the higher dosage of EPO in the HDP-low cohort could simply reflect the compromised clinical state of these subjects, as supported by the higher Couchoud score in HDP low responders. Nevertheless, the EPO receptor plays a significant role in B cell development and activation, and ubiquitin is involved in B cell activation and B cell receptor regulation (68, 69).

Our feature selection identified an early gene signature at day 0 and day 7 that could discriminate between healthy and HDP cohorts, as well as between HDP-high and -low responder groups. These genes generally exhibited opposite expression patterns in the healthy control and HDP-low individuals. At day 0, a set of 32 genes differentiates our three cohorts, of those genes, 10 had previously been identified as differentially expressed in studies on kidney diseases. This kidney disease-associated profile could support the observation that the HDP-low cohort includes individuals with a worse clinical state compared with the other cohorts, as indicated by their higher Couchoud risk score before vaccination. Among the 15-gene signature of day 7, 4 genes were expressed in mast cells and these were overexpressed in the HDP-low cohort. Increased plasmatic histamine, correlated with an increase in *HDC* (Histidine Decarboxylase), has been associated with an increase in mast cells in a subgroup of HDP (70).

Our study has some limitations: the small sample size, which may limit the generalizability of our findings; the inclusion of participants with a wide age range, which was not matched between cohorts and showed a statistically significant difference between healthy and fragile subjects; the classification of HDP-high and -low responders based on antibody levels measured right after the second vaccine dose, that may not represent the peak of the acute immune response or long term memory (19).

In conclusion, our study identified genes and pathways that were differentially expressed in HDP-low compared to HC and HDP-high at baseline and 7 days after primary vaccination, potentially related with their impaired immune response the BNT162b2 mRNA vaccine. HDP-low represented 45% of the HDP cohort and were characterized by alterations in genes associated with pre-existing chronic inflammation, cell cycle, EPO-related pathways, reduced expression of B cell-related genes at baseline, and genes potentially correlated with renal disease, while further differences emerged in genes involved in B cell regulation and survival at day 7. Our analyses also highlighted the importance of conducting a characterization of immunocompromised cohorts to identify predictive biomarkers of vaccine immunogenicity and dysregulated pathways affecting vaccine responsiveness.

## Data Availability

The datasets presented in this study can be found in online repositories. The names of the repository/repositories and accession number(s) can be found below: https://www.ncbi.nlm.nih.gov/geo/query/acc.cgi?acc=GSE265975, GSE265975.
