# Supplementary material for: Transcriptomic analysis after SARS-CoV-2 mRNA vaccination reveals a specific gene signature in low-responder hemodialysis patients
Source: Front Immunol. 2025 Apr 30;16:1508659. doi: 10.3389/fimmu.2025.1508659 (PMC12075225; doi:10.3389/fimmu.2025.1508659)
Supplement: Supplementary file 1 [file Table1.pdf]

Supplementary table 1. Population characteristics at baseline. Medians and IQR, or counts and frequencies are reported. *P-value <0.05

	HDP (n=20)	HC (n=9)	P-value
**Age, years**	69.5 (53.25-78)	40 (32-57.5)	**0.006***
**Male sex**	15 (75%)	4 (44%)	0.20
**Dialysis age, years**	7.5 (3.25-10.75)	-	-
**Comorbidity, yes/no**	20 (100%)	-	-
**Neoplasia**	5 (25%)	-	-
**Diabetes**	6 (30%)	-	-
**Heart disease**	8 (40%)	-	-
**Immunosuppressive therapy**	2 (10%)	-	-
