# Supplementary material for: Transcriptomic analysis after SARS-CoV-2 mRNA vaccination reveals a specific gene signature in low-responder hemodialysis patients
Source: Front Immunol. 2025 Apr 30;16:1508659. doi: 10.3389/fimmu.2025.1508659 (PMC12075225; doi:10.3389/fimmu.2025.1508659)
Supplement: Supplementary file 2 [file Table2.docx]

**Supplementary Table 2. Cluster separation in the UMAP dimensionality-reduced space**. A K-means clustering was performed on UMAP dimensionality-reduced space and the results were compared with actual group labels. For each day a contingency matrix is reported with accuracy as a metric.

| **Time point** | **Cluster** | **HDP^1^ Low responders** | **HDP^1^ High responders** | **HC^1^** | **Accuracy** |
| --- | --- | --- | --- | --- | --- |
| **Day 0** | Low responders | 7 | 1 | - | **0.71** |
|  | High responders | 2 | 8 | 3 |  |
|  | HC | - | 2 | 5 |  |
|  |  |  |  |  |  |
| **Day 7** | Low responders | 5 | 1 | - | **0.77** |
|  | High responders | 1 | 5 | - |  |
|  | HC | - | 2 | 4 |  |
|  |  |  |  |  |  |
| **Day 21** | Low responders | 5 | 2 | 1 | **0.61** |
|  | High responders | 1 | 5 | - |  |
|  | HC | 1 | 4 | 7 |  |
|  |  |  |  |  |  |
| **Day 28** | Low responders | 7 | 4 | - | **0.64** |
|  | High responders | 1 | 6 | 4 |  |
|  | HC | 1 | - | 5 |  |

^1^ HDP=Hemodialysis patients

^2^ HC= Healthy Controls
