# Supplementary material for: Transcriptomic analysis after SARS-CoV-2 mRNA vaccination reveals a specific gene signature in low-responder hemodialysis patients
Source: Front Immunol. 2025 Apr 30;16:1508659. doi: 10.3389/fimmu.2025.1508659 (PMC12075225; doi:10.3389/fimmu.2025.1508659)
Supplement: Supplementary file 5 [file Table5.pdf]

Supplementary table 5. Comparison between High and Low Responders HDP. Medians and IQR, or counts and frequencies are reported. BV: before vaccination. *P value <0.05

	HDP-high (n=11)	HDP-low (n=9)	P-value
**Age, years**	73 (52 - 75)	68 (51 - 88)	0.63
**Male sex**	9 (81%)	6 (67%)	0.61
**Dialysis age, years**	7 (3-11)	9 (3.5-10.5)	0.87
**EPO BV (IU/week)**	2000 (1000-5000)	8000 (3000-24000)	**0.04***
**EPO six months BV (IU/week)**	2000 (0-8000)	10000 (4000-21000)	**0.03***
**Comorbidity, yes/no**	11 (100%)	9 (100%)	1.00
**Neoplasia**	1 (9%)	4 (44%)	0.12
**Diabetes**	1 (9%)	5 (56%)	0.05
**Heart disease**	4 (36%)	4 (44%)	1.00
**Immunosuppressive therapy**	0 (0%)	2 (22%)	0.19
**Couchoud score**	3.0 (1.9 – 4.2)	12.8 (6 – 14.2)	**0.03***
