# Supplementary material for: Transcriptomic analysis after SARS-CoV-2 mRNA vaccination reveals a specific gene signature in low-responder hemodialysis patients
Source: Front Immunol. 2025 Apr 30;16:1508659. doi: 10.3389/fimmu.2025.1508659 (PMC12075225; doi:10.3389/fimmu.2025.1508659)
Supplement: Supplementary file 6 [file DataSheet1.pdf]

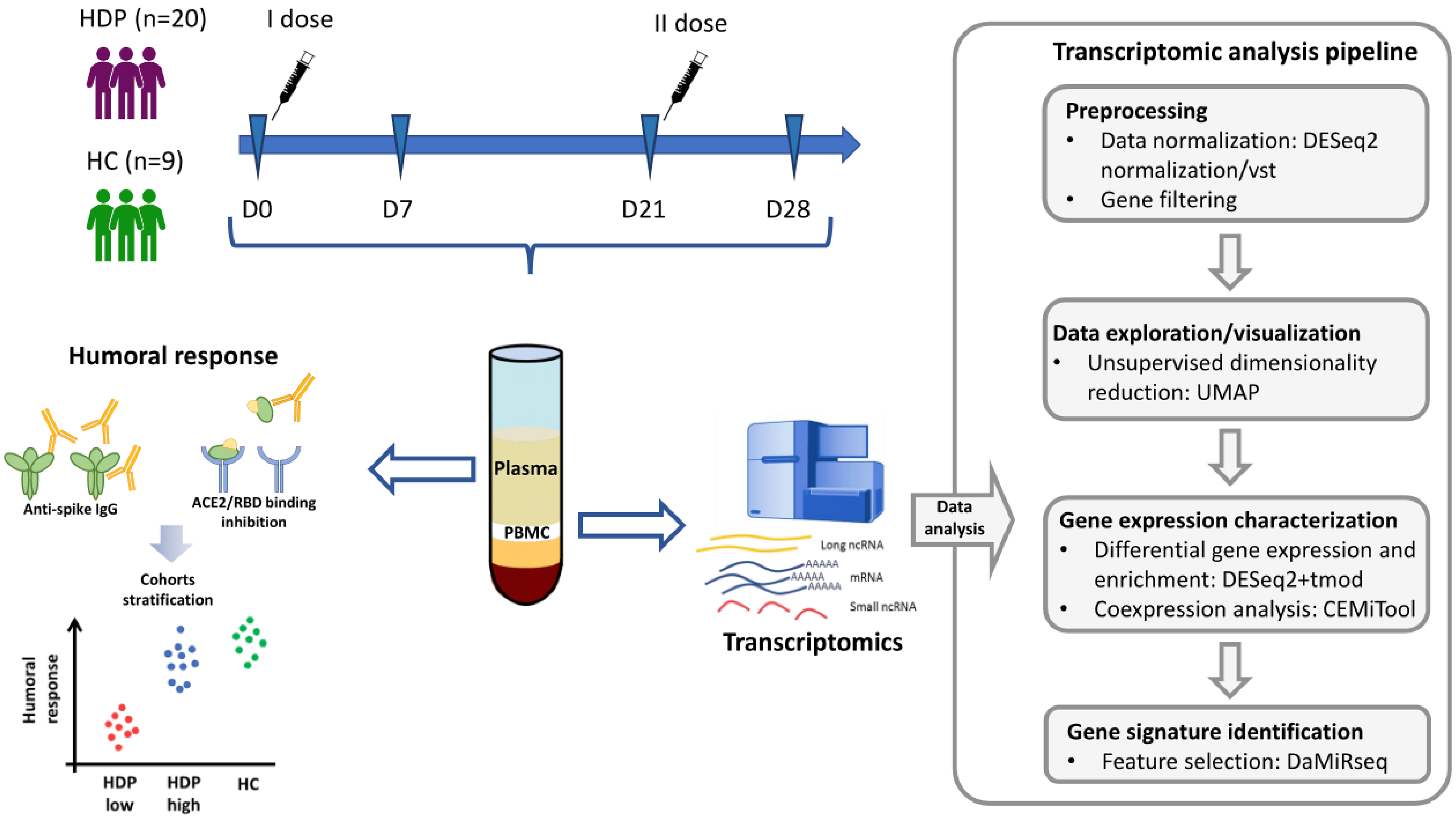

Supplementary figure 1. Study design and workflow. A total of 20 HDP and 9 HC who received two doses of BNT162b2 SARS-CoV-2 mRNA vaccine were enrolled in this study. Blood samples were collected at baseline (D0), days 7 and 21 after the first dose, and day 28 (7 days after the second dose). The humoral response was assessed at day 28, measuring anti-spike IgG levels and ACE2/RBD binding inhibition to stratify the HDP cohort. PBMCs were used for transcriptomic profiling. The transcriptomic analysis pipeline included different analysis approaches, including unsupervised dimensionality reduction, differential gene expression analysis, co-expression analysis, and gene signature identification.
