# Supplementary material for: Transcriptomic analysis after SARS-CoV-2 mRNA vaccination reveals a specific gene signature in low-responder hemodialysis patients
Source: Front Immunol. 2025 Apr 30;16:1508659. doi: 10.3389/fimmu.2025.1508659 (PMC12075225; doi:10.3389/fimmu.2025.1508659)
Supplement: Supplementary file 7 [file DataSheet2.pdf]

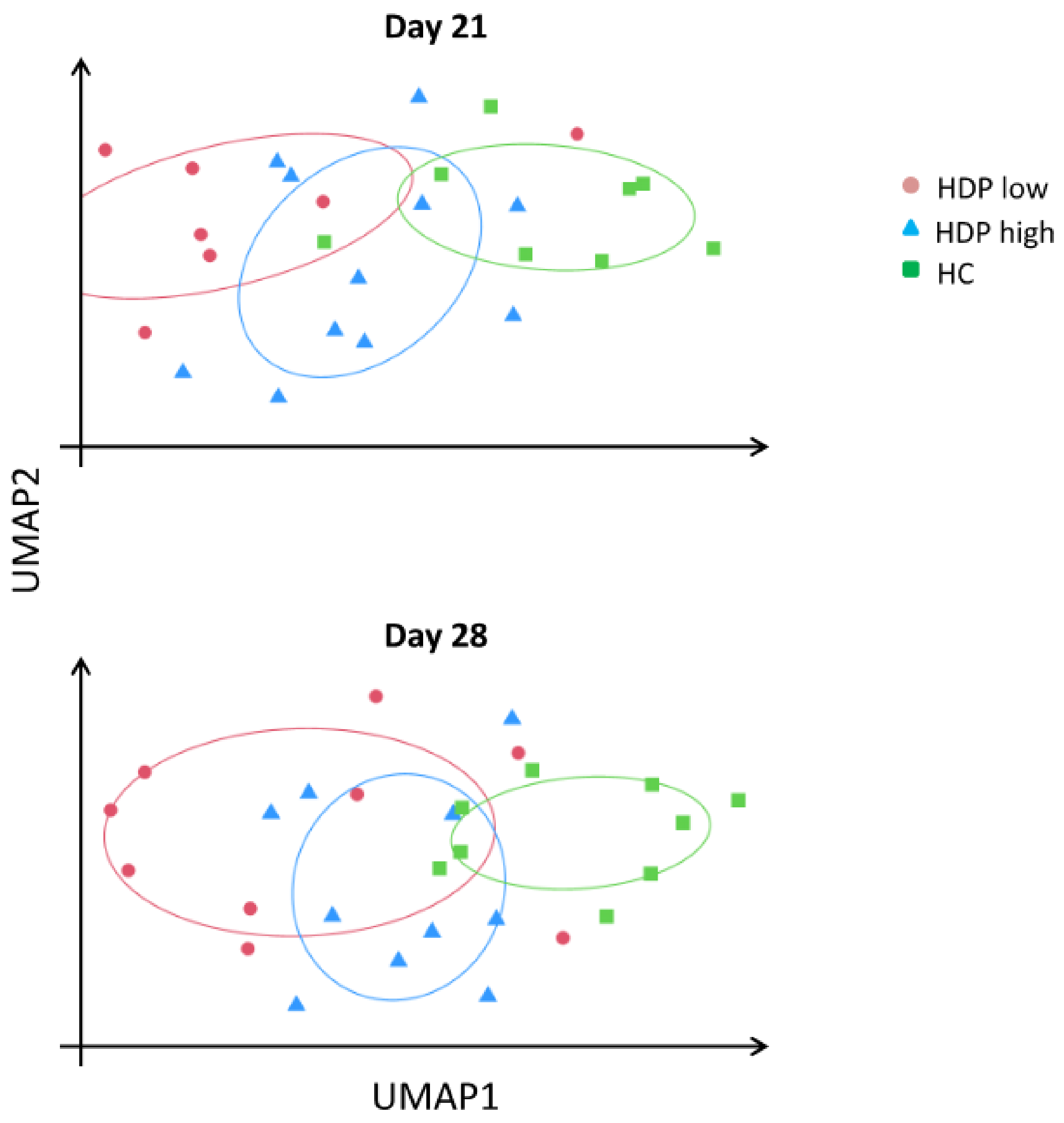

Supplementary figure 2. UMAP dimensionality reduction at day 21 and day 28. Normalized and z-score transformed gene counts were dimensionally reduced using the UMAP (Uniform Manifold Approximation and Projection) algorithm. Each dot represents a sample in the two new surrogate dimensions (UMAP1 and UMAP2). Each color represents an experimental group. Colored ellipses represented 75% confidence intervals.
