# Supplementary material for: Transcriptomic analysis after SARS-CoV-2 mRNA vaccination reveals a specific gene signature in low-responder hemodialysis patients
Source: Front Immunol. 2025 Apr 30;16:1508659. doi: 10.3389/fimmu.2025.1508659 (PMC12075225; doi:10.3389/fimmu.2025.1508659)
Supplement: Supplementary file 8 [file DataSheet3.pdf]

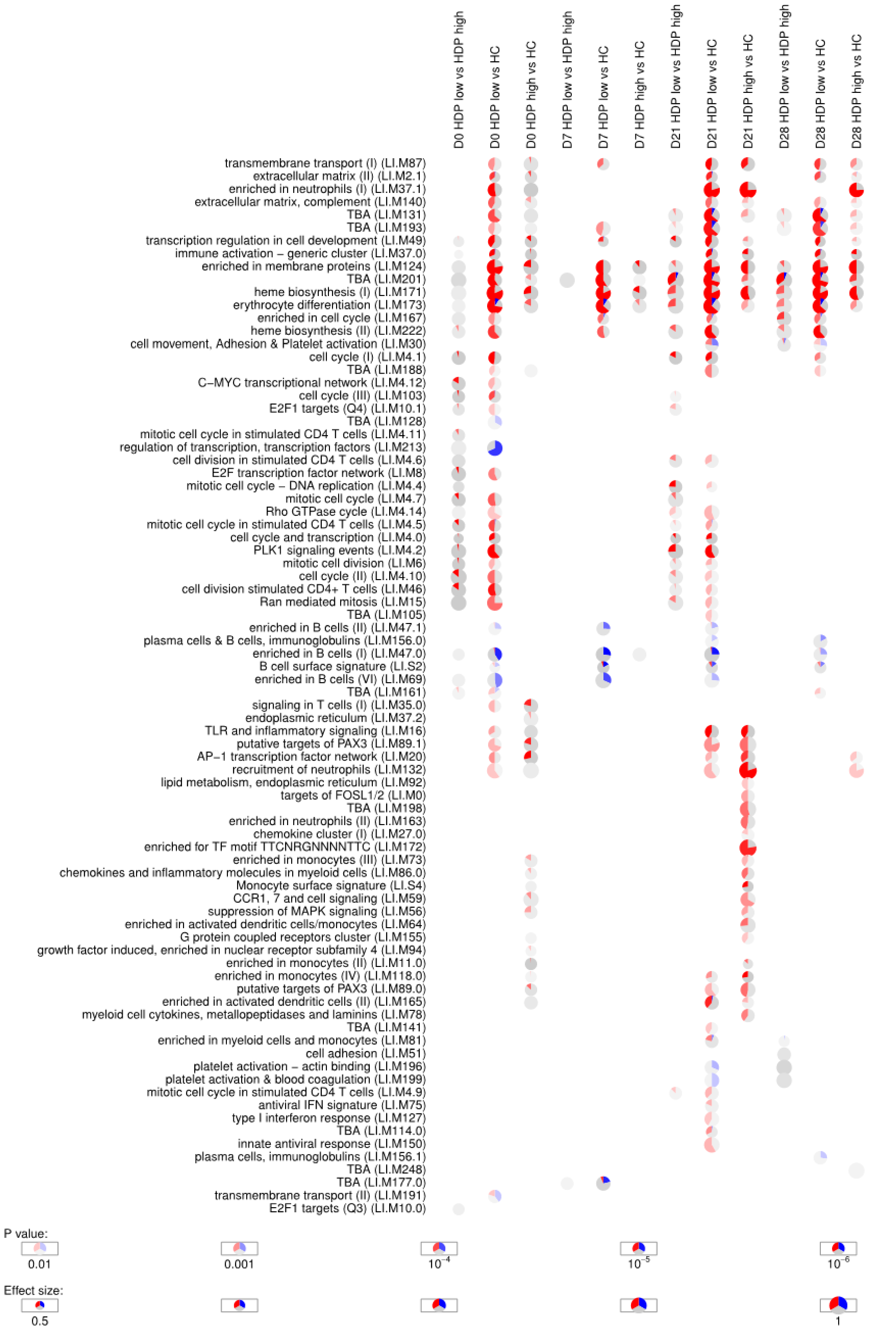

Supplementary figure 3. Enrichment analysis. Activation of blood transcription modules by BNT162b2 vaccination in healthy controls (HC) and hemodialysis patients stratified in high- and low-responders groups (HDP-high and HDP-low, respectively). Each column represents a comparison. Activation of modules was tested using tmod CERNO test on the false discovery rate (FDR)-ranked lists of genes generated by DESeq2. Rows indicate different blood transcription modules, which were significantly (FDR < 0.01) activated in at least one comparison. Each module is represented as a pie plot in which the proportion of significantly upregulated and downregulated genes is shown in red and blue, respectively. The grey portion of the pie represents genes that are not significantly differentially regulated according to DGE analysis. The significance of module activation is proportional to the color intensity of the pie, while the effect size (Area Under the Curve) is proportional to its size.
