# Supplementary material for: Transcriptomic analysis after SARS-CoV-2 mRNA vaccination reveals a specific gene signature in low-responder hemodialysis patients
Source: Front Immunol. 2025 Apr 30;16:1508659. doi: 10.3389/fimmu.2025.1508659 (PMC12075225; doi:10.3389/fimmu.2025.1508659)
Supplement: Supplementary file 9 [file DataSheet4.pdf]

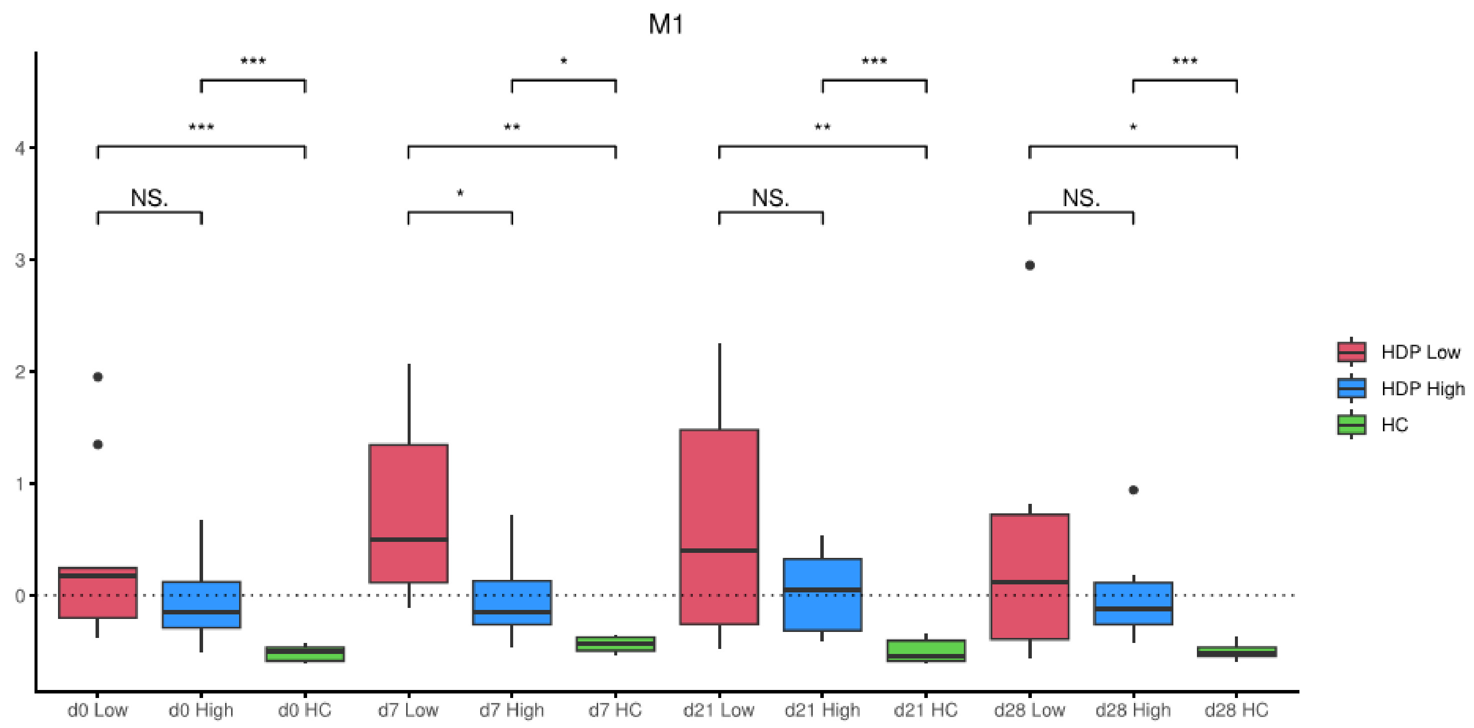
M1
***
*
***
***
***
**
**
*
NS.
*
NS.
NS.
4
3
2
1
0
d0 Low
d0 High
d0 HC
d7 Low
d7 High
d7 HC
d21 Low
d21 High
d21 HC
d28 Low
d28 High
d28 HC
HDP Low
HDP High
HC

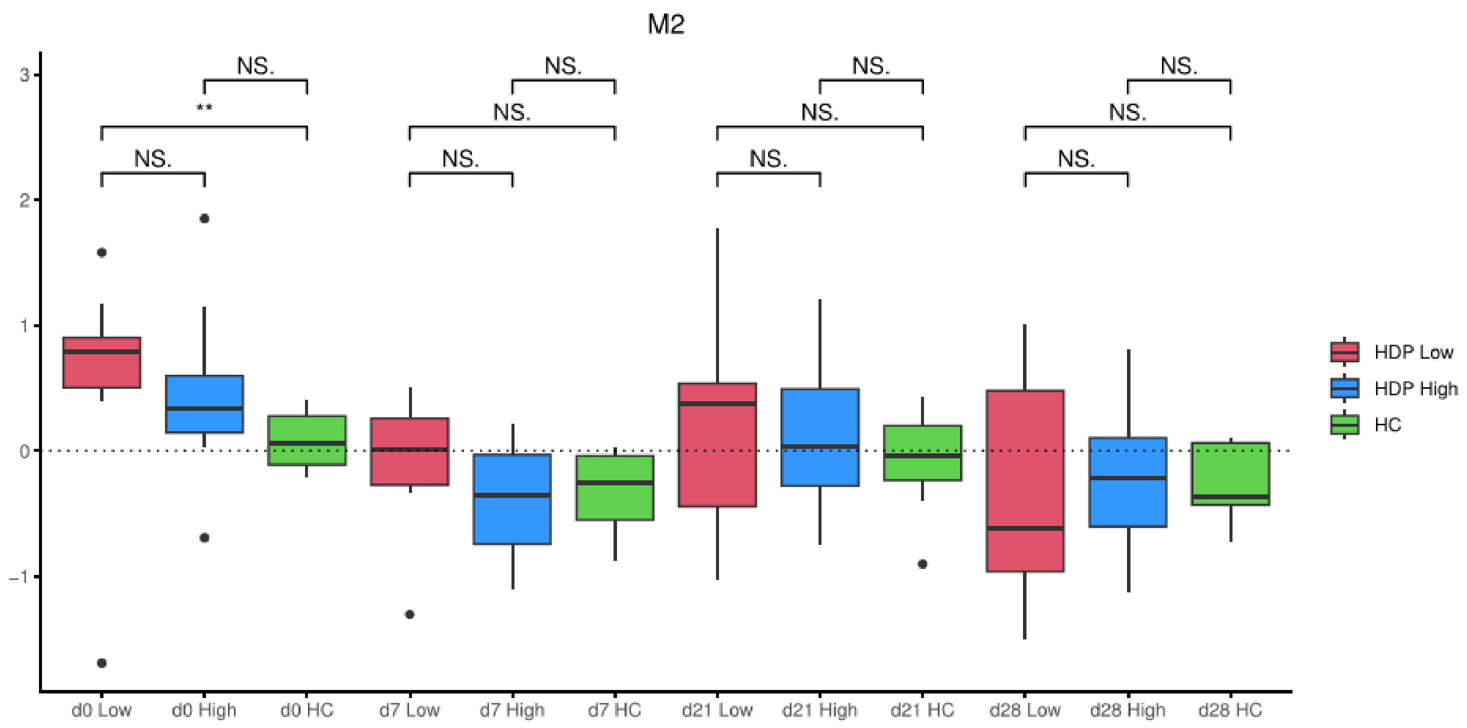
M2
NS.
**
NS.
NS.
NS.
NS.
NS.
NS.
NS.
NS.
NS.
NS.
NS.
3
2
1
0
−1
d0 Low
d0 High
d0 HC
d7 Low
d7 High
d7 HC
d21 Low
d21 High
d21 HC
d28 Low
d28 High
d28 HC
HDP Low
HDP High
HC

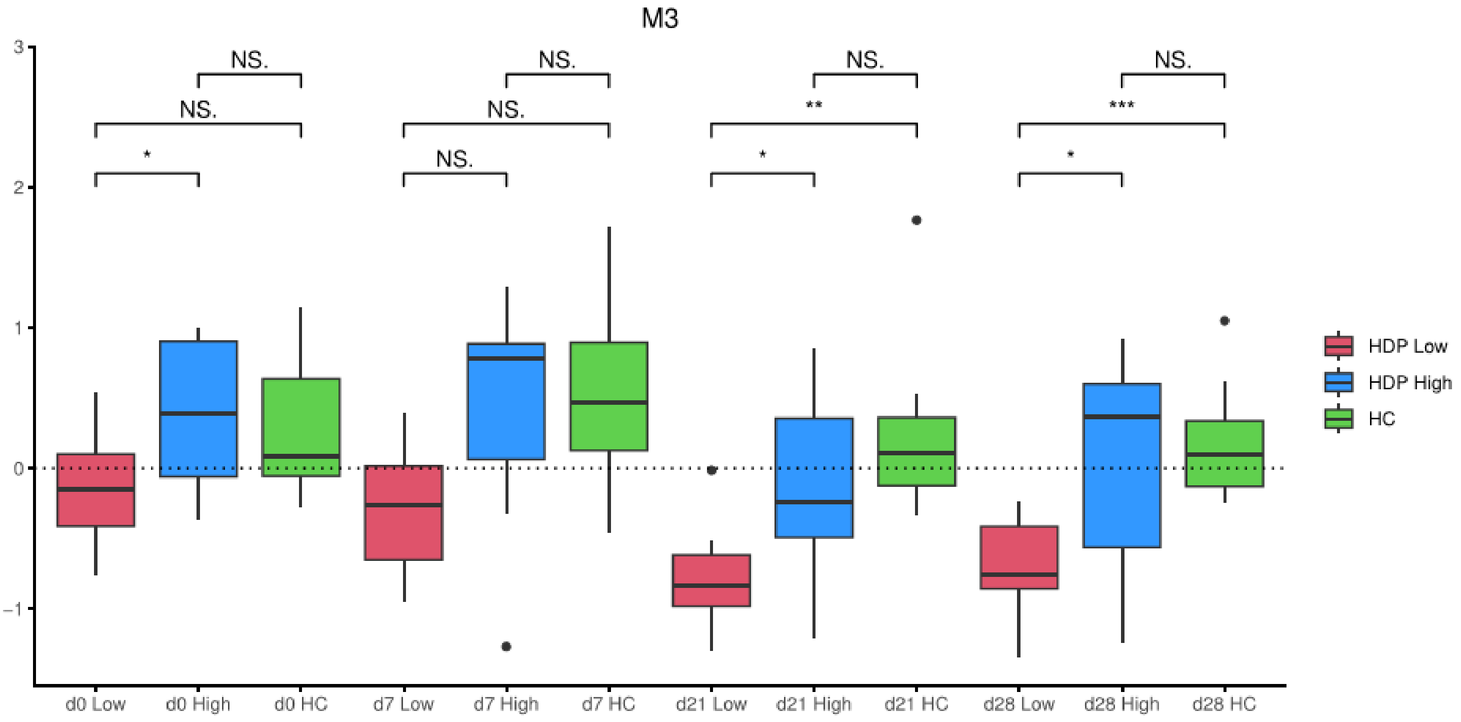
M3
NS.
NS.
*
NS.
NS.
NS.
NS.
**
*
NS.
***
*
3
2
1
0
−1
d0 Low
d0 High
d0 HC
d7 Low
d7 High
d7 HC
d21 Low
d21 High
d21 HC
d28 Low
d28 High
d28 HC
HDP Low
HDP High
HC

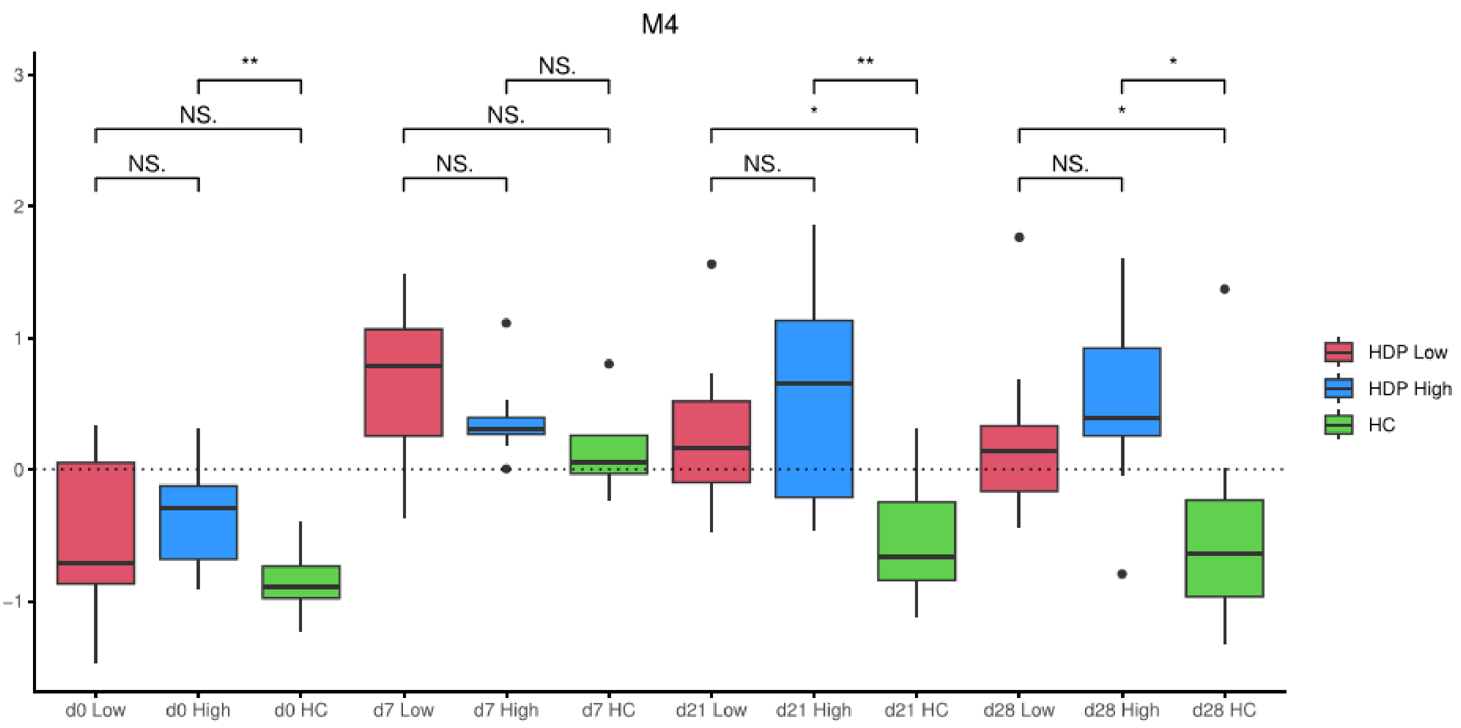

M4
**
NS.
**
*
NS.
NS.
*
NS.
NS.
NS.
NS.
NS.
3
2
1
0
−1
d0 Low
d0 High
d0 HC
d7 Low
d7 High
d7 HC
d21 Low
d21 High
d21 HC
d28 Low
d28 High
d28 HC
HDP Low
HDP High
HC

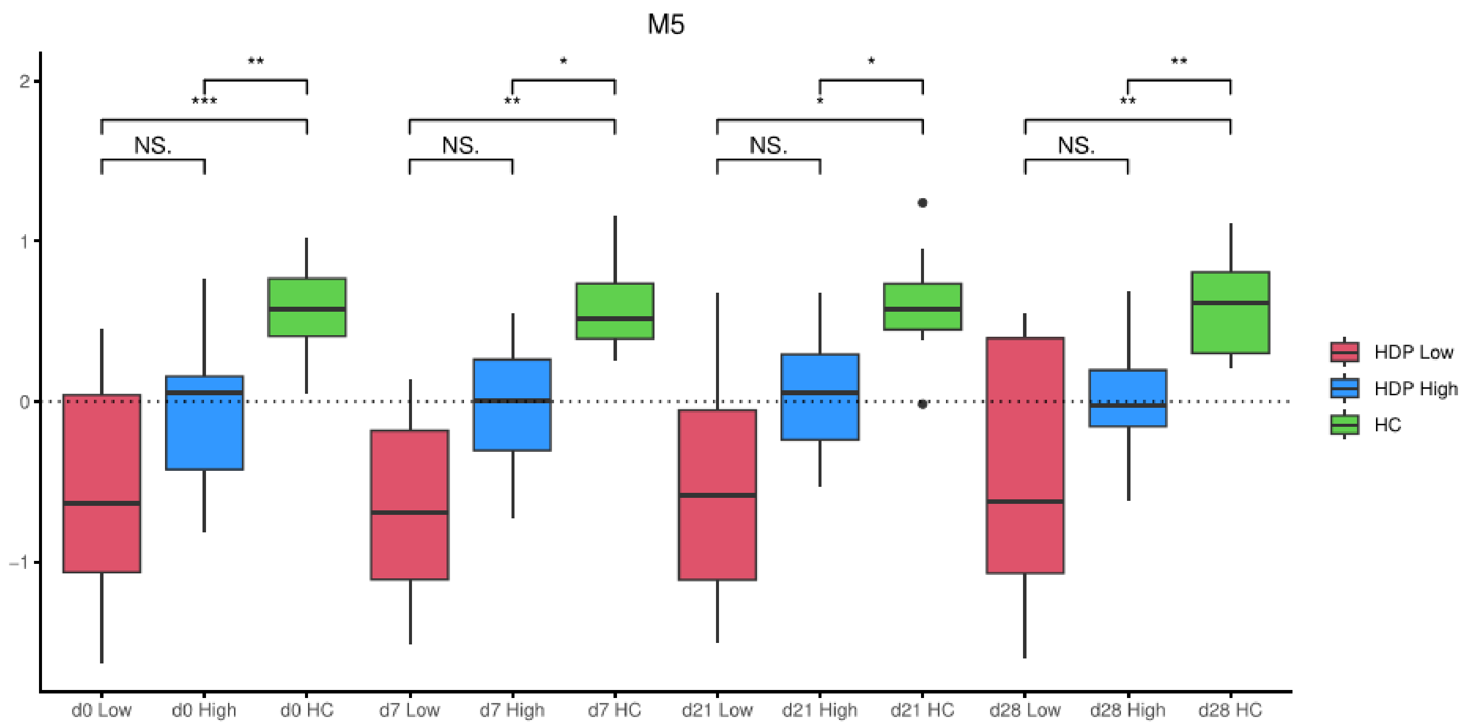

M5
2
1
0
−1
NS.
NS.
NS.
NS.
***
**
**
*
*
*
**
**
d0 Low
d0 High
d0 HC
d7 Low
d7 High
d7 HC
d21 Low
d21 High
d21 HC
d28 Low
d28 High
d28 HC
HDP Low
HDP High
HC

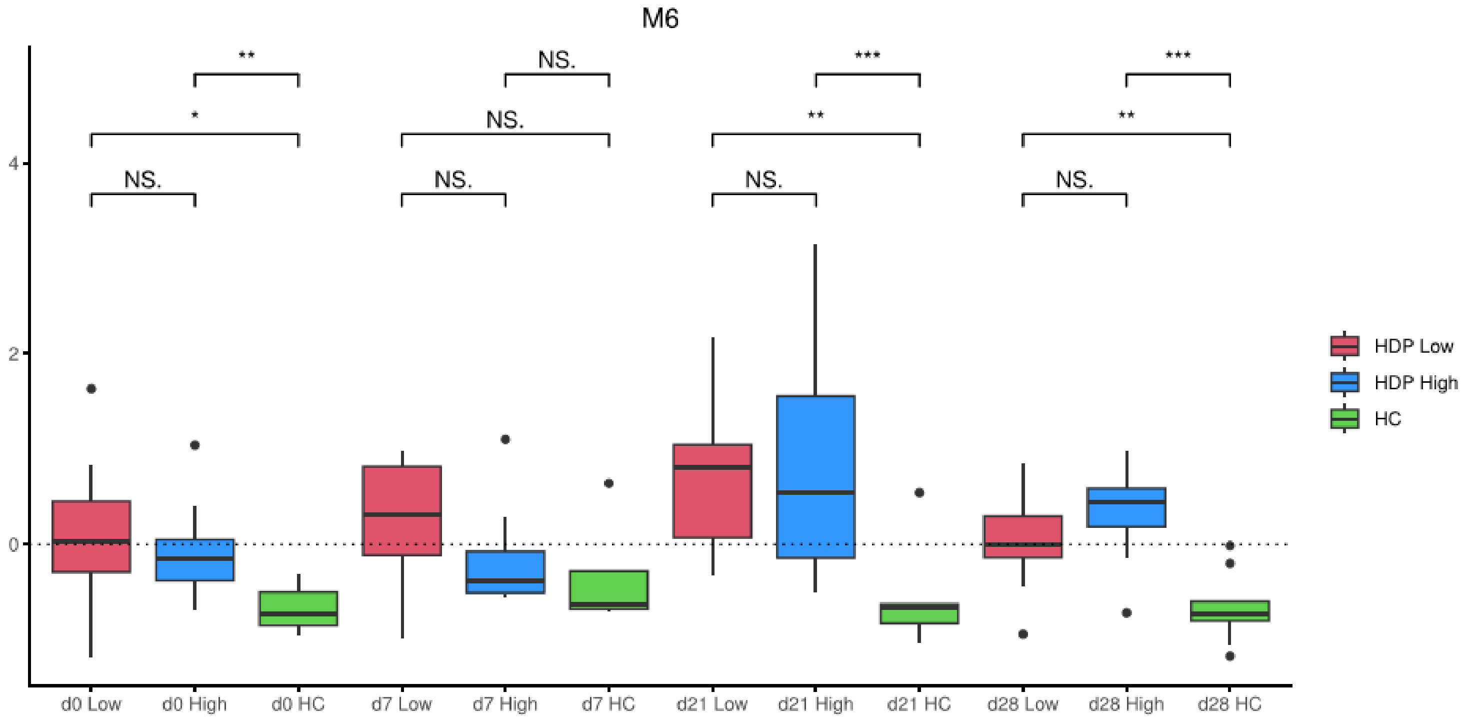
M6
**
***
***
NS.
*
NS.
**
**
NS.
NS.
NS.
NS.
4
2
0
HDP Low
HDP High
HC
d0 Low
d0 High
d0 HC
d7 Low
d7 High
d7 HC
d21 Low
d21 High
d21 HC
d28 Low
d28 High
d28 HC

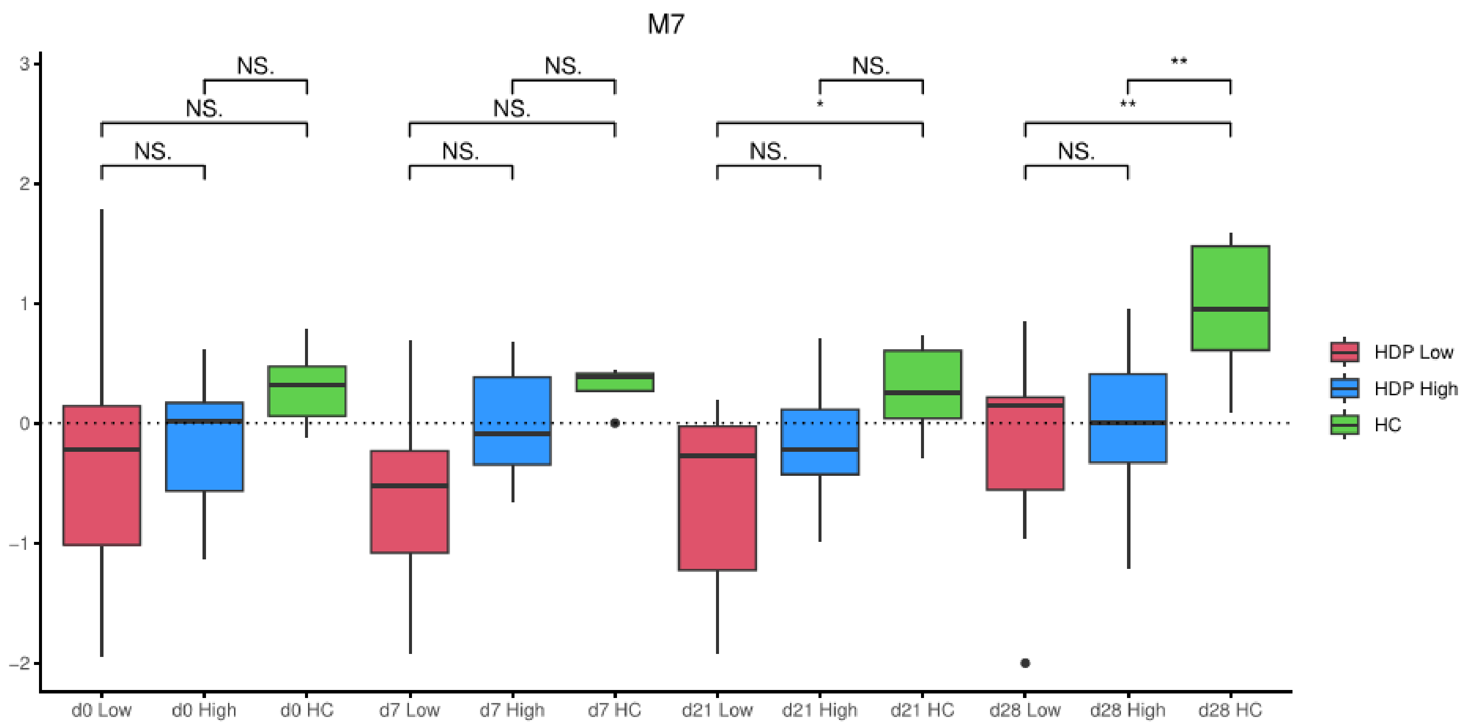
M7
NS.
NS.
NS.
NS.
NS.
NS.
NS.
*
NS.
**
**
NS.
3
2
1
0
−1
−2
d0 Low
d0 High
d0 HC
d7 Low
d7 High
d7 HC
d21 Low
d21 High
d21 HC
d28 Low
d28 High
d28 HC
HDP Low
HDP High
HC

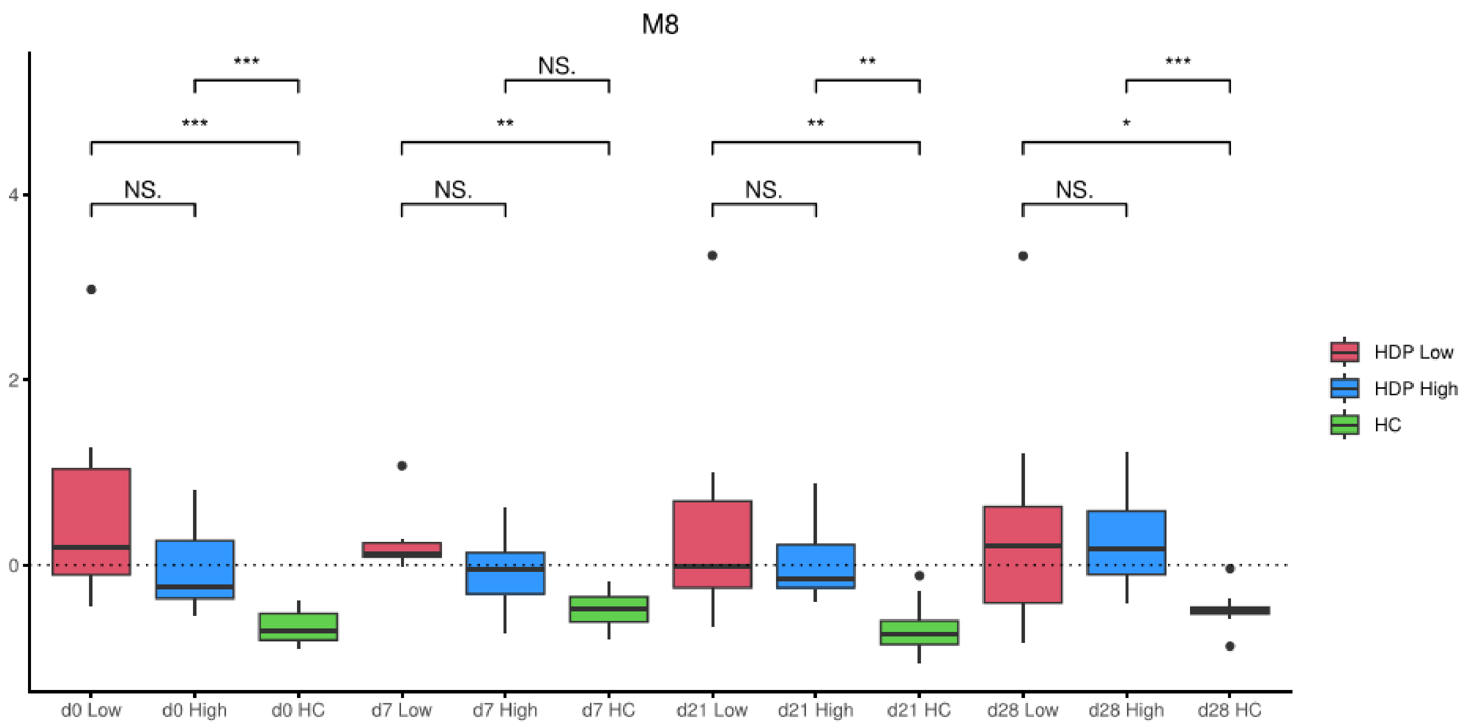
M8
***
NS.
**
***
***
**
**
*
NS.
NS.
NS.
NS.
4
2
0
HDP Low
HDP High
HC
d0 Low
d0 High
d0 HC
d7 Low
d7 High
d7 HC
d21 Low
d21 High
d21 HC
d28 Low
d28 High
d28 HC

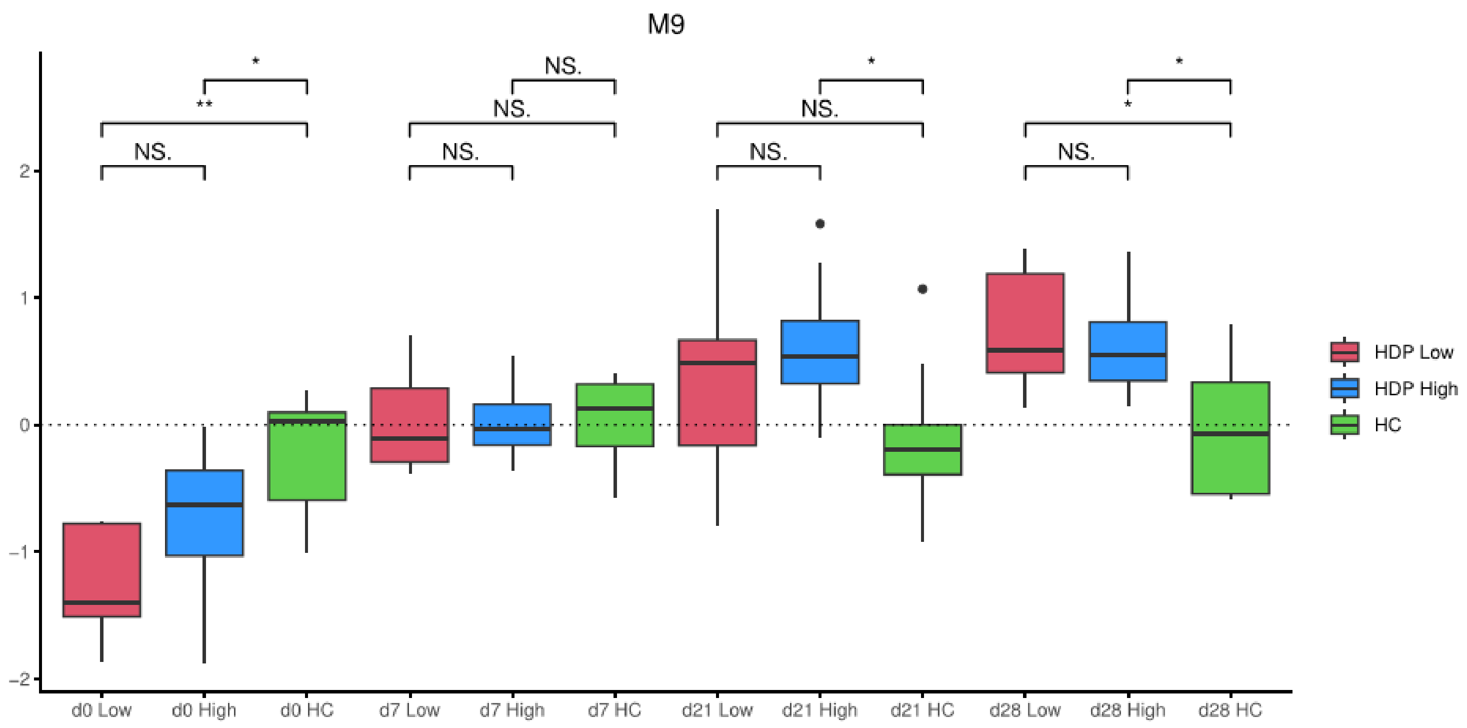

M9
NS.
**
*
NS.
NS.
NS.
NS.
NS.
*
NS.
NS.
*
*
2
1
0
−1
−2
d0 Low
d0 High
d0 HC
d7 Low
d7 High
d7 HC
d21 Low
d21 High
d21 HC
d28 Low
d28 High
d28 HC
HDP Low
HDP High
HC

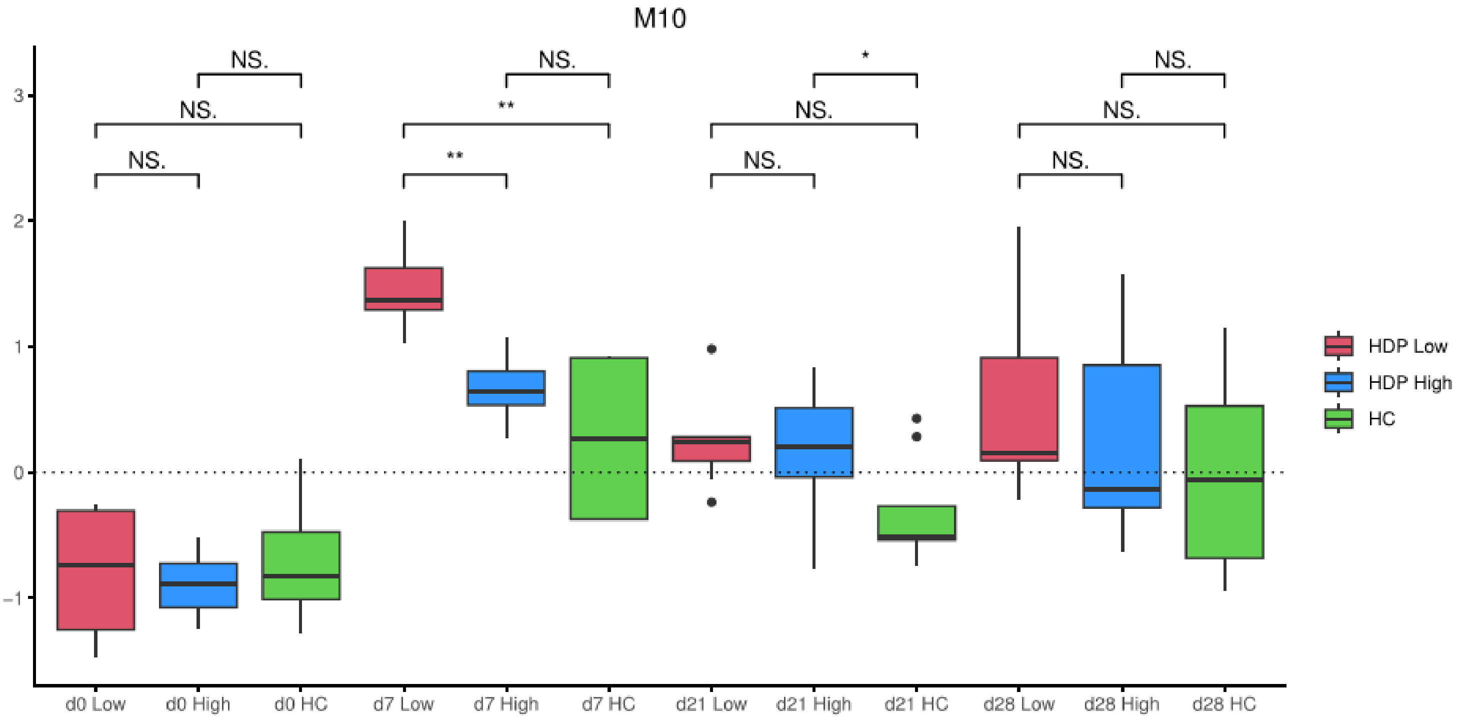
M10
NS.
NS.
NS.
NS.
**
**
*
NS.
NS.
NS.
NS.
NS.
3
2
1
0
−1
d0 Low
d0 High
d0 HC
d7 Low
d7 High
d7 HC
d21 Low
d21 High
d21 HC
d28 Low
d28 High
d28 HC
HDP Low
HDP High
HC

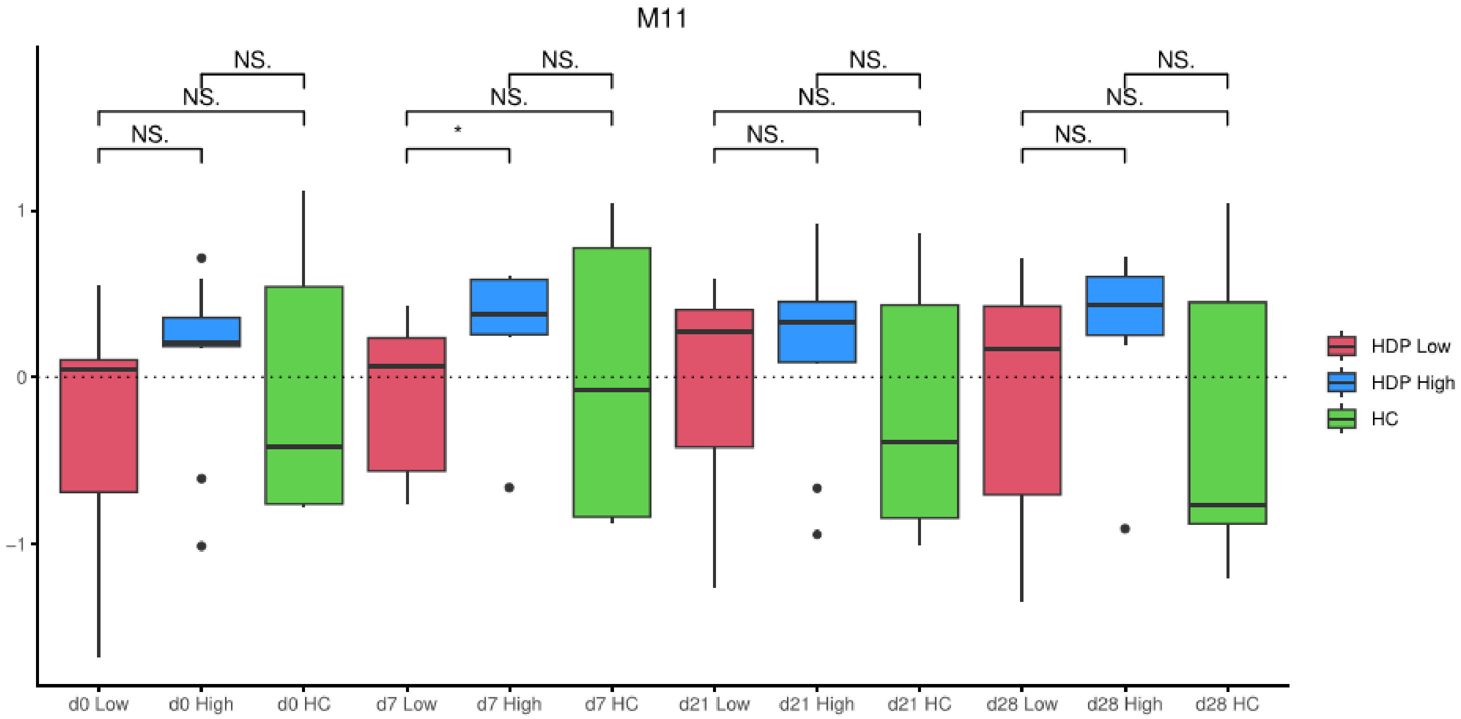
M11
NS.
NS.
NS.
*
1
0
−1
d0 Low
d0 High
d0 HC
d7 Low
d7 High
d7 HC
d21 Low
d21 High
d21 HC
d28 Low
d28 High
d28 HC
HDP Low
HDP High
HC

Supplementary figure 4. CEMiTool analysis. A CEMiTool analysis was performed on the normalized gene counts to group genes with similar expression and characterize different expression patterns. Eleven modules of co-expressed genes were identified and each panel represents a module. The module score, calculated as the mean z-score of the genes included in the module, in different groups at different time points is represented as a box and whiskers plot where the box represents the IQR, the horizontal line represents the median value, and whiskers represent higher and lower values. Individual module score values of outliers are reported as dots. Dashed lines represent the mean value for each module. The non-parametric Mann-Whitney Test was used to assess significant differences (*p-value<0.05, **p-value<0.01, ***p-value<0.001).
