# Supplementary material for: Transcriptomic analysis after SARS-CoV-2 mRNA vaccination reveals a specific gene signature in low-responder hemodialysis patients
Source: Front Immunol. 2025 Apr 30;16:1508659. doi: 10.3389/fimmu.2025.1508659 (PMC12075225; doi:10.3389/fimmu.2025.1508659)
Supplement: Supplementary file 10 [file DataSheet5.pdf]

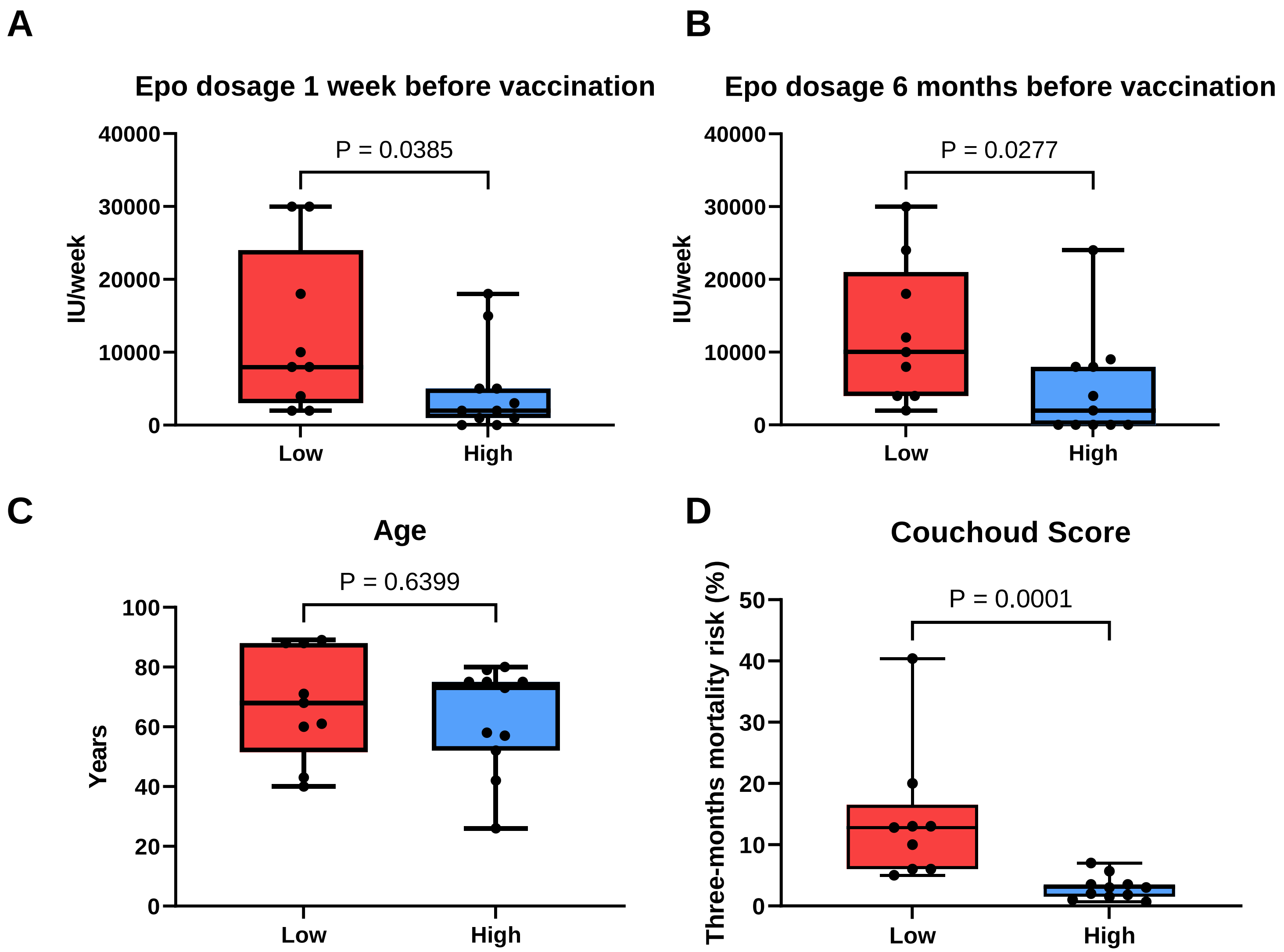

Supplementary figure 5. Statistical test on clinical and demographic parameters. Data showed as box and whiskers plot, with each dot representing a samples, were tested to assess differences of pre-vaccination weekly erythropoietin dosage (in A), the average erythropoietin dosage in the 6 months prior to vaccination (in B), age (in C), and three-months mortality risk calculated with Couchoud method (in D), in hemodialysis patients low- and high-responders (in red and blue, respectively). The non-parametric Mann-Whitney Test was used to assess significant differences.
